# Revisiting the comparative phylogeography of unglaciated eastern North America: 15 years of patterns and progress

**DOI:** 10.1002/ece3.8827

**Published:** 2022-04-19

**Authors:** Rachel Ann Lyman, Christine E. Edwards

**Affiliations:** ^1^ Ecology, Evolution, and Population Biology Program Washington University in St. Louis St. Louis Missouri USA; ^2^ Center for Conservation and Sustainable Development Missouri Botanical Garden St. Louis Missouri USA

**Keywords:** biogeographic discontinuity, comparative phylogeography, divergence time estimation, ecological niche modeling, pleistocene glaciation, unglaciated eastern North America

## Abstract

In a landmark comparative phylogeographic study, “Comparative phylogeography of unglaciated eastern North America,” Soltis et al. (*Molecular Ecology*, 2006, 15, 4261) identified geographic discontinuities in genetic variation shared across taxa occupying unglaciated eastern North America and proposed several common biogeographical discontinuities related to past climate fluctuations and geographic barriers. Since 2006, researchers have published many phylogeographical studies and achieved many advances in genotyping and analytical techniques; however, it is unknown how this work has changed our understanding of the factors shaping the phylogeography of eastern North American taxa. We analyzed 184 phylogeographical studies of eastern North American taxa published between 2007 and 2019 to evaluate: (1) the taxonomic focus of studies and whether a previously detected taxonomic bias towards studies focused on vertebrates has changed over time, (2) the extent to which studies have adopted genotyping technologies that improve the resolution of genetic groups (i.e., NGS DNA sequencing) and analytical approaches that facilitate hypothesis‐testing (i.e., divergence time estimation and niche modeling), and (3) whether new studies support the hypothesized biogeographic discontinuities proposed by Soltis et al. (*Molecular Ecology*, 2006, 15, 4261) or instead support new, previously undetected discontinuities. We observed little change in taxonomic focus over time, with studies still biased toward vertebrates. Although many technological and analytical advances became available during the period, uptake was slow and they were employed in only a small proportion of studies. We found variable support for previously identified discontinuities and identified one new recurrent discontinuity. However, the limited resolution and taxonomic breadth of many studies hindered our ability to clarify the most important climatological or geographical factors affecting taxa in the region. Broadening the taxonomic focus to include more non‐vertebrate taxa, employing technologies that improve genetic resolution, and using analytical approaches that improve hypothesis testing are necessary to strengthen our inference of the forces shaping the phylogeography of eastern North America.

## Graphical abstract

We surveyed the phylogeographical literature published between 2006 and 2019 and evaluated how both the field of phylogeography and our understanding of the major phylogeographical forces shaping taxa in unglaciated eastern North America has changed over time. Our review is important in that it provides not only an updated understanding of the historical factors shaping the phylogeography of species in the region but also because it highlights the fact that studies suffer from a notable lack of taxonomic breadth and that only a small proportion are employing technologies that can help improve resolution and hypothesis testing ability of phylogeographic studies. Our article highlights the need for improved taxonomic diversity in phylogeographical studies to broaden our inference of the generality of the forces shaping the geographical patterns of genetic variation across species. We also conclude that it is imperative that future studies begin to employ technologies and analytical approaches that will improve their hypothesis‐testing ability.

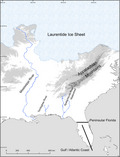

## 1 INTRODUCTION

Phylogeography is a field that bridges research from the fields of population genetics, phylogenetics, population ecology, and historical biogeography, addressing how historical biogeographical and climatological factors shape population genetic structure (Bermingham & Avise, 1986). To understand the broader biogeographical forces shaping patterns of genetic variation across communities and ecosystems, Bermingham and Avise (1986) laid out the theoretical groundwork for comparative phylogeography, which compares phylogeographic patterns of multiple species with overlapping distributions to test for a shared common history. Avise (2000) reviewed all published intraspecific phylogeographic studies to search for commonalities among the patterns found across species and identified possible common forces shaping the patterns of genetic variation across the landscape. Nearly two decades later, Soltis et al. (2006) explored the comparative phylogeography of unglaciated eastern North America, a geologically and ecologically complex region with a high degree of diversity and endemic species. Based on published phylogeographic studies that employed molecular data in plants, animals, fungi, and protists, the goal of the study was to synthesize the commonalities among these studies, uncover broad phylogeographic patterns in the biota of the region, and to decipher the biogeographical and geological factors that may have given rise to these patterns.

**FIGURE 1:**
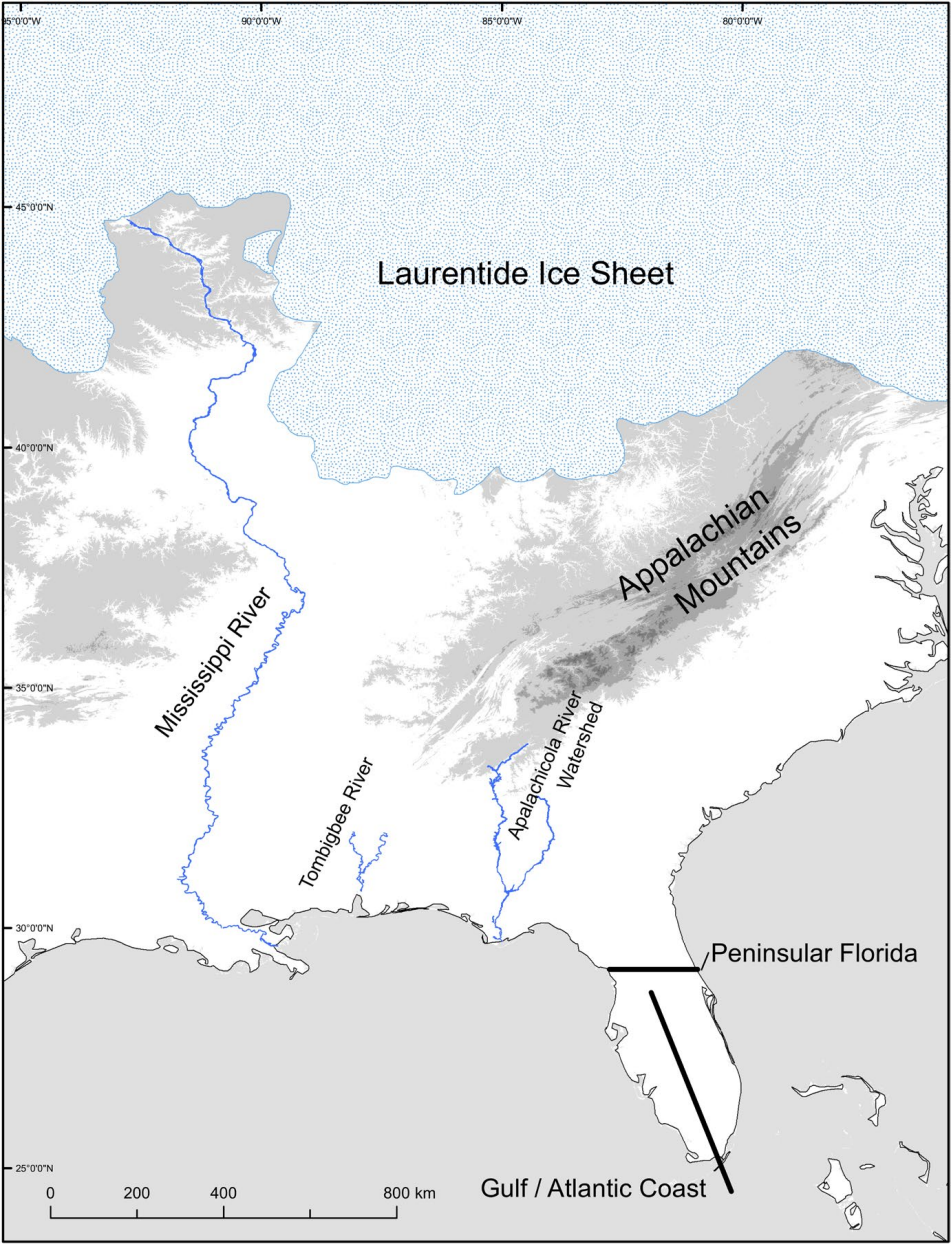
Hypothesized biogeographic discontinuities in eastern North America

By proposing a series of phylogeographical patterns and hypotheses against which phylogeographical studies could be compared and tested, Soltis et al. (2006) became pivotal to phylogeographic work in eastern North America. Based on shared patterns of phylogeography and analysis of the climatological and geographical history in unglaciated eastern North America, Soltis et al. (2006) outlined the following six recurrent phylogeographic discontinuities (i.e., a distinct geographic pattern in the distribution of alleles and relationships between them, often characterized by a sharp geographic boundary between genetic groups that corresponds to major geographic features): (1) Atlantic Coast/Gulf Coast, (2) Apalachicola River, (3) Tombigbee River, (3) Appalachian Mountains, (4) Mississippi River, and (5) the Appalachian Mountain/Apalachicola River and Mississippi River discontinuity (Figure 1; Table 1). An additional proposed pattern was Pleistocene refugia occupied by species located just south of the Laurentide Ice Sheet (Figure 1; Table 1).

**TABLE 1:** Summary of discontinuities in this study

Discontinuity	Pattern	Divergence time assumption	Cause of discontinuity	Limitations
Atlantic Ocean/Gulf Coast (Maritime)	Distinct Atlantic Ocean and Gulf Coast lineages; break occurs somewhere along the eastern side of southern Florida peninsula	Post‐Pleistocene (<0.0117 mya)	Inhospitable climate, ocean currents, and habitat preventing gene flow between the coasts	No clearly defined geographical break
Apalachicola River (Riverine)	Distinct lineages on east and west sides of the Apalachicola River	Pleistocene (2.58–0.0117 mya)	Expansion of southern river drainages leading to the Gulf of Mexico during Pleistocene interglacial periods	Many other rivers (i.e., Tombigbee River, Chattahoochee River) in the region
Appalachian Mountains (Terrestrial)	Distinct lineages on east and west sides of the Appalachian Mountains	post mid‐Miocene to late‐Pleistocene (14.2 mya–0.0117 mya)	Mountain range dates over 480 million years; result of multiple cycles of geological uplift, weathering, and erosion; last uplift occurred during the mid‐Miocene; may also occur because of Pleistocene refugia east and west of the Appalachians	Confusion with Apalachicola River barrier
Mississippi River (Riverine)	Distinct lineages on east and west sides of the Mississippi River	Pleistocene (2.58–0.01127 mya)	Altering of the flow, size, and course of the river and expansion of floodplain forests due to flooding during Pleistocene interglacial periods	Frequent changes to the course and size during Pleistocene glacial cycles
Apalachicola River/ Appalachian Mountains and Mississippi River (Terrestrial/Riverine)	Three distinct lineages: 1) west of the Mississippi, 2) east of the Mississippi River and west of the Apalachicola River, and 3) east of the Apalachicola River	Pleistocene (2.58–0.0117 mya)	See individual barriers	Too broad of a distribution; Too many potential geologic and climatic causal factors
Laurentide Ice Sheet (Terrestrial)	Distinct lineages found north of the southernmost range (39°N) of the Laurentide Ice Sheet	Last glacial advance (115–12 kya)	Peak of last glacial maximum led to its southernmost expansion, with a proposed Pleistocene refugia located just south of the southern extent of the ice sheet. The unique haplotypes found north of the southernmost extent of the Laurentide ice dispersed northward in response to warming after the Pleistoce	Difficult to distinguish from other potential barriers (i.e., Appalachian Mountains); Lack of genetic differentiation
Florida Peninsula (Terrestrial)	Distinct lineage confined to peninsular Florida (east of the Suwannee River)	mid‐Miocene to late‐Pleistocene (14.2–0.0117 mya)	Glacial cycles causing the submerging and exposure of the larger Florida platform	Wide range of potential divergence time estimates

All the discontinuities outlined by Soltis et al. (2006) implicate climatological forces and/or physical barriers as the most likely causal factors in shaping how the genetic variation of species is structured across the landscape, many of which occurred during the Pleistocene (Figure 1; Table 1). For example, the riverine discontinuities (i.e., the Apalachicola River, Tombigbee River, and Mississippi River) were proposed to be caused by climatological warming during the Pleistocene, which melted glaciers and expanded the rivers, adjacent floodplains, and floodplain forests, such that they became major barriers to gene flow between populations occurring on either side of the river (Table 1). Similarly, the Appalachian Mountain discontinuity is generally attributed to populations occupying two distinct Pleistocene refugia on opposite sides of the Appalachians (Soltis et al., 2006; Table 1). The Appalachian Mountain/Apalachicola River and Mississippi River discontinuity is a combined discontinuity involving three proposed Pleistocene refugia: one east of the Appalachian Mountains/Apalachicola River, one between the Appalachian Mountains/Apalachicola River and the Mississippi River, and one to the west of the Mississippi River. Finally, the refugia south of the Laurentide Ice Sheet is less of a discontinuity and more of a genetic pattern, whereby unique haplotypes are restricted to Northern areas formerly covered by the Laurentide Ice Sheet, potentially indicating that they persisted in low‐density populations in close proximity to the Laurentide Ice Sheet during the Pleistocene and dispersed northward in response climate warming after the Pleistocene (Soltis et al., 2006).

In addition to outlining these phylogeographical discontinuities based on comparative analysis of early phylogeographical studies, Soltis et al. (2006) found evidence of a notable taxonomic bias in study organisms, with around 60% of the studies focused on vertebrates (particularly fish), 21% focused on plants, and only 3% focused on Fungi (Soltis et al., 2006); it is unclear whether studies published since Soltis et al. (2006) have focused on a greater breadth of taxa. It is also unclear whether adding additional studies (which may focus on a more diverse range of taxa) provides additional support for the hypothesized discontinuities proposed by Soltis et al. (2006) or whether they support other shared discontinuities that were not found previously due to a lack of taxonomic breadth in the literature at the time of the study.

Since the Soltis et al. (2006) paper, numerous advances in genotyping technologies have been introduced that increase the resolution of phylogeographic analyses. Whereas early studies generally employed limited genetic data, such as sequences of one or a few mtDNA loci or data from a few allozyme loci (Avise et al., 1984), many newer studies now have the ability to employ genetic technologies that are more high‐throughput and provide greater resolution of genetic groups and phylogenetic relationships. Currently, technology has developed to the point where studies may employ dozens of microsatellites (Converse et al., 2017; Hodel, Segovia‐Salcedo, et al., 2016; Schrey et al., 2011; Williams et al., 2008), whole organellar genomes (Farrington et al., 2017), thousands of genome‐wide nuclear markers (Duvernell et al., 2019; Grabowski et al., 2014; Hamlin & Arnold, 2014; Martin et al., 2016; Zhou et al., 2018), or whole‐genome resequencing (Bourgeois et al., 2019). However, although many advancements have been made in genotyping technologies over the past 15 years, it is unclear the extent to which phylogeographical studies have begun to employ these technologies (but see Morris & Shaw, 2018), whether these approaches have improved the resolution of phylogeographic discontinuities in phylogeographical studies, and whether the potential increased resolution of genetic groups and phylogeographical discontinuities has changed our overall understanding of the relative importance of phylogeographic discontinuities in eastern North America.

Accompanying the improvements in genotyping technologies is the development of analytical techniques to analyze molecular data and test phylogeographical hypotheses. One important hypothesis‐testing tool is software to date the divergences between lineages. One option is BEAST, which uses Bayesian analysis and the multispecies coalescent to reconstruct time‐calibrated phylogenies. BEAST is most commonly employed to test the timing of divergences among multiple species in a clade and requires fossil data or other calibration points to accurately infer divergence times. Other programs, such as DIYABC, IM, ∂a∂I, and fastsimcoal (Cornuet et al., 2008; Excoffier & Foll, 2011; Gutenkunst et al., 2009; Sethuraman & Hey, 2016) have been developed to test hypotheses about the demographic history of populations, which have dramatically improved the ability to conduct phylogeographical hypothesis testing. These programs use genetic data to evaluate and test the relative likelihood of evolutionary scenarios involving the order and timing of divergences among lineages, genetic bottlenecks, gene flow among lineages, and changes in effective population size (Cornuet et al., 2014). Historical ecological niche modeling is another type of computational approach that can be used to help infer the climatological forces that have affected phylogeographic patterns; its main use in phylogeography is to infer the distribution of suitable habitat of a species in the past (Guisan & Thuiller, 2005; Peterson, 2006; Peterson et al., 1999). Another related approach to infer how past climate shaped current patterns of genetic structure is iDDC (He et al., 2013), which can be incredibly powerful for determining how past climate shaped the divergences among lineages. However, the extent to which phylogeographical studies have begun to employ these new analytical approaches to aid in hypothesis testing in is unknown. Also unclear is whether the increased ability to resolve historical scenarios provided by these new analytical technologies has changed our understanding of phylogeographic discontinuities in eastern North America.

In the present study, we revisited the comparative phylogeography of unglaciated eastern North America by surveying the phylogeographic studies published since Soltis et al. (2006). The goals of this study were (1) to understand whether the taxonomic focus of phylogeographical studies and the previously observed taxonomic bias toward vertebrate studies has changed over time, (2) to evaluate the adoption of technologies that can improve the resolution and hypothesis‐testing ability of phylogeographic studies, namely next‐generation DNA sequencing (NGS) technologies, divergence time estimation, and ecological niche modeling, (3) to assess the relative strength of evidence for the biogeographic discontinuities proposed by Soltis et al. (2006) and whether specific groups of taxa show common discontinuities by investigating the proportion of taxa or studies that exhibited each discontinuity, and (4) whether new studies showed evidence for additional phylogeographic discontinuities not found previously by Soltis et al. (2006).

## 2 METHODS

### 2.1 Survey of the literature

A database of studies conducted since the publication of Soltis et al. (2006) was compiled using Web of Science searches. The search was restricted to papers published between 2007 and 2019. We conducted the following searches: (1) all papers citing Soltis et al. (2006); and (2) searches using the topic search terms “North America” combined with one of the following terms: “phylogeograph*” “landscape genetics” “comparative phylogeograph*”. The literature searches resulted in 3,550 papers combined. Duplicate entries, review articles, studies lacking genetic data, studies lacking any metrics of statistical support for phylogeographical patterns, or those not conducted at a relevant taxonomic level (i.e., comparisons between genera) or in a relevant geographic region were removed. The review of each paper individually narrowed to the number of papers to 184.

For each paper, the following were recorded: (1) study species; (2) whether analyses were conducted at the inter‐ or intraspecific level (studies comparing subspecies were categorized as intraspecific regardless of how they were described in the paper); (3) the molecular techniques employed (i.e., microsatellites, Sanger sequencing, NGS approaches, etc.); (4) the phylogeographic discontinuity observed (i.e., Mississippi River) and the strength of statistical support for the discontinuity (see below); (5) whether ecological niche modeling was used to test phylogeographic hypotheses; and (6) whether the divergence time was estimated, how it was estimated, and the date of divergence, which was categorized into stage/age according to The ICS International Chronostratigraphic Chart (Martin et al., 2013) (Appendix S1; Table S1). Several approaches were used to understand the strength of statistical support for the phylogeographic divergence. For analyses that used bootstrap or Bayesian posterior probability support values, we considered genetic divergences to be significant if they had a minimum support value of 70/0.95 respectively. In the case of AMOVA, we considered it to be significant if the p‐value of among‐group variance was less than 0.001. STRUCTURE results were assessed based on the extent to which geographic groups were assigned to distinct genetic groups and the amount of admixture present between geographical groups (with significant admixture categorized as <80% assignment to a majority cluster).

## 3 RESULTS

**FIGURE 2:**
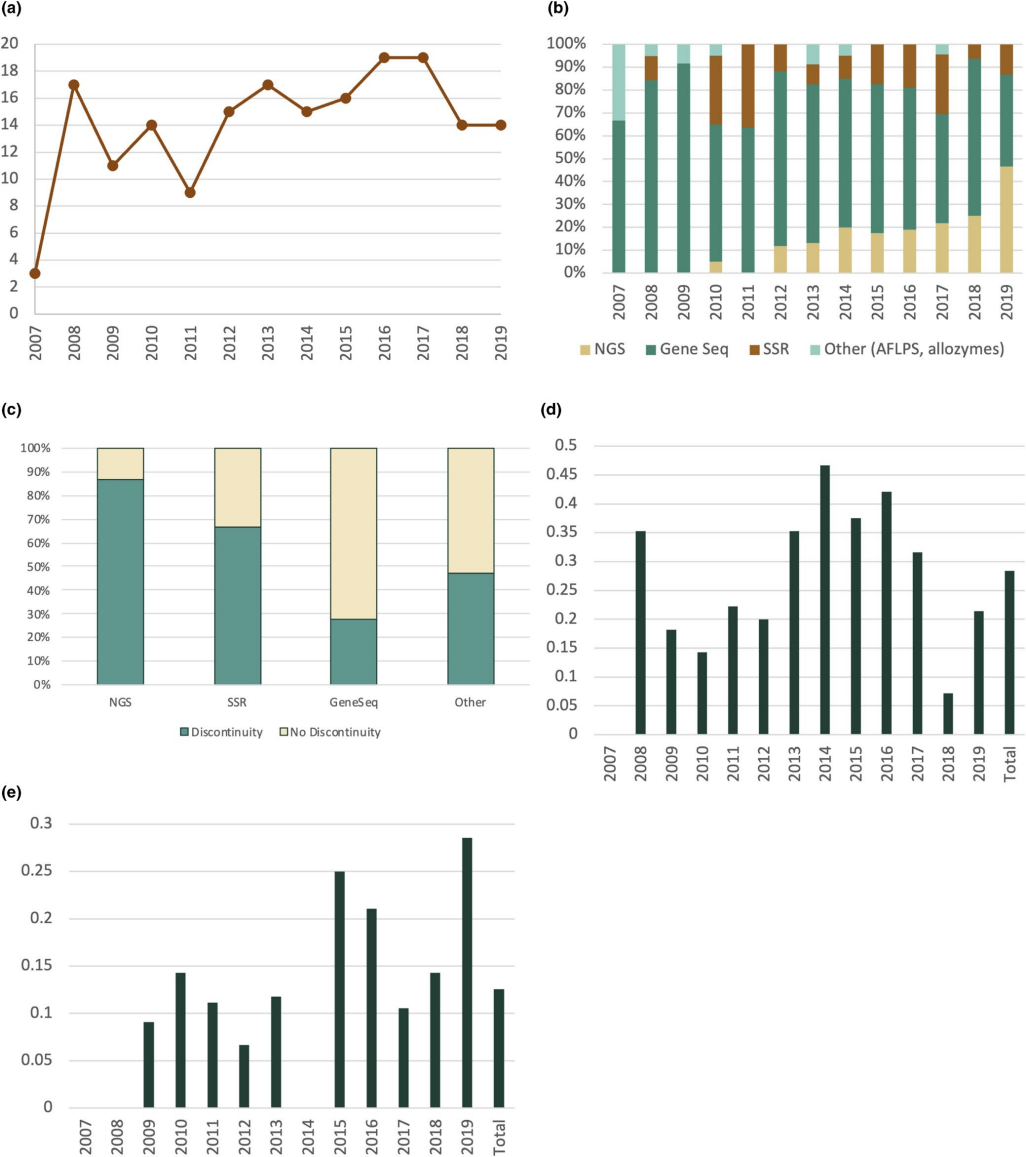
(a) Total number of studies surveyed by publication year; (b) Stacked proportion of molecular markers used in studies surveyed by publication year; (c) Stacked proportion showing whether a discontinuity was detected by molecular marker; (d) Proportion of studies surveyed that estimated divergence times by publication year; (e) Proportion of studies surveyed that used Ecological Niche Modeling (ENM) by publication year

The search criteria identified a list of 184 papers conducted on relevant taxa within the study region between 2007 and 2019 (Appendix S1; Table S1). The number of papers published per year ranged from 3 in 2007 to 19 in 2016 and 2017 (Figure 2a).

In the survey of the literature by taxon, the majority of studies (95 studies, or 52% of total) focused on vertebrates, with most studies conducted in fish (30 studies, or 16% of total), reptiles (30 studies, or 16% of total), and amphibians (15 studies, or 8% of total), and only a small number of studies focused on birds or mammals, representing 10 (5% of the total) and 11 (6% of total) studies, respectively (Table 2). In total, 56 studies focused on plants (30%), 53 of which focused on angiosperms (95% of plant studies) (Table 2). We identified 31 studies that focused on invertebrates (17% of total), which were heavily skewed toward arthropods (22 studies, or 71% of invertebrate studies). Only two studies focused on fungi (1.09%). Overall, these values were largely consistent with those found in Soltis et al. (2006), except that a smaller overall proportion of recent studies focused on fish (24% vs. 16%) and a greater proportion focused on plants (13% vs. 29%) and arthropods (4% vs. 12%) (Table 2).

**TABLE 2:** Number of phylogeographical studies and the total species surveyed by taxonomic group, with the percentage of overall studies indicated in parentheses

Taxon	Number of studies—Soltis et al. (2006)	Number of species—from Soltis et al. (2006)	Number of studies—present study	Number of species—present study
Arthropod	5 (4%)	4 (3%)	22 (12%)	70 (38%)
Crustacean	7 (5%)	10 (7%)	2 (1%)	2 (1%)
Mollusk	7 (5%)	9 (6%)	6 (3%)	12 (7%)
Bryozoan	1 (0.7%)	1 (0.7%)	0	0
Hydrozoan	1 (0.7%)	1 (0.7%)	0	0
Total Invertebrate	21 (15%)	25 (18%)	31 (17%)	84 (46%)
Amphibian	12 (9%)	12 (9%)	15 (8%)	38 (21%)
Bird	16 (11%)	16 (11%)	10 (5%)	10 (5%)
Fish	33 (24%)	43 (31%)	30 (16%)	93 (51%)
Mammal	7 (5%)	7 (5%)	11 (6%)	14 (8%)
Reptile	16 (11%)	20 (14%)	30 (16%)	48 (26%)
Total Vertebrate	84 (60%)	98 (70%)	95 (52%)	203 (110%)
Angiosperm	18 (13%)	15 (11%)	53 (29%)	131 (71%)
Gymnosperm	5 (4%)	5 (4%)	2 (1%)	2 (1%)
Algae, Mosses, Liverworts	7 (5%)	5 (3%)	1 (0.5%)	1 (0.5%)
Total Plant	**30 (21%)**	**25 (18%)**	**56 (30%)**	**134 (72%)**
Fungi	4 (3%)	3 (2%)	2 (1%)	13 (7%)
Overall total	140	151	184	434

Note

Bold values represent the combined number and percentage of studies in broad taxonomic groups, composed of the smaller subgroups listed immediately above.

In the survey of the literature by genotyping approach, the majority of studies (146, 78%) used DNA sequences of one or a few selected loci (e.g., a limited number of mitochondrial loci in animals or plastid loci in plants) to understand the phylogeography of eastern North American taxa, and the proportion of studies employing DNA sequences of one or a few loci was largely consistent over time (Figure 2b). A small but consistent proportion of studies each year employed microsatellites, whereas studies employing allozymes or AFLPs were infrequent and decreased over time, with only a single study published utilizing these markers since 2014 (Figure 2b). The overall proportion of studies employing NGS approaches, including restriction site‐associated DNA sequencing (RADseq), genotyping‐by‐sequencing (GBS), Illumina targeted‐amplicon sequencing (TAS) of many loci, or Illumina sequencing of whole organellar genomes, represents only a small proportion of the total number of studies (34 studies, or 19% of total). However, the first NGS‐based studies were published in 2010 and the proportion has gradually increased over time, such that nearly half of the studies published in 2019 employed NGS technologies (Figure 2b). In terms of the ability of markers to resolve phylogeographic discontinuities, a much greater proportion of studies employing NGS technologies identified a discontinuity compared to those employing microsatellites, Sanger sequencing of a few loci, or other methods (Figure 2c).

A relatively low proportion of phylogeographic papers (52 studies, or 28% of total) from this region dated the divergences among lineages. The number of studies employing divergence time estimation ranged from zero papers in 2007, to a peak of between 6 and 8 per year between 2013–2017 (32%–47% of studies per year; Figure 2d). BEAST was the program most frequently used to estimate divergence times. Although a few studies employed DIYABC, it was only used to test the order of divergences but not the divergence date (Martin et al., 2013). Of the studies that calculated divergence times, a large proportion focused on reptiles (15, or 29%), angiosperms (12, or 23%), and fish (10, or 19%), whereas only a few studies dated divergence times in arthropods, birds, amphibians, and mammals.

The number and proportion of studies that utilized ecological niche modeling (ENM) to test phylogeographical hypotheses in addition to genetic analysis was relatively low (23 studies, or 12.5% of total), but the use of this approach increased over time. In three earlier years (2007, 2008, and 2014), no published papers utilized ENM (Figure 2e). In the remaining years, the number of studies utilizing ENM ranged between one and four, with recent years showing the greatest proportion of studies employing ENM, including 2015 (25% of the studies in that year), 2016 (21% of the studies in that year) and 2019 (29% of the studies in that year) (Figure 2e).

### 3.1 Survey of the literature by discontinuity

A total of 138 of 184 papers (75%) found significant evidence for a discontinuity in the geographic patterns of genetic diversity (Table 3). Most discontinuities identified in the present study matched those identified previously by Soltis et al. (2006), with the most common being the Mississippi River discontinuity (46 studies, or 25% of the total), followed by the Atlantic/Gulf Coast discontinuity (31 studies, or 17% of total), the Appalachian Mountains (23 studies, or 12.5% of the total), the Apalachicola River (19 studies, or 10% of total) (Table 3), and south of the Laurentide Ice Sheet (17 studies, or 9% of total). We found little support for the Apalachicola River/Appalachian Mountains and Mississippi River discontinuity, which was only observed in three studies (1.64%). We also observed two recurrent phylogeographic patterns that were not categorized by Soltis et al. (2006): peninsular Florida and smaller riverine systems (see discussion for detailed descriptions of each). The peninsular Florida discontinuity, which refers to a line running roughly from around Jacksonville, FL, directly west to the Gulf of Mexico that separates peninsular Florida from continental North America (Figure 1), was one of the most common (36 studies, or 20% of total). Relative to Soltis et al. (2006), a greater proportion of recent papers found evidence for the Mississippi River discontinuity (9% vs. 25%) and Florida peninsula discontinuity (3% vs. 20%), whereas fewer found evidence for the Apalachicola River discontinuity (20% vs. 10%).

**TABLE 3:** Summary of the phylogeographic discontinuities (see Table 1) observed by taxonomic group in Soltis et al. (2006), the present study, and overall

Discontinuity	Results of Soltis et al. (2006)	Results of present study	Total
Atlantic Ocean/Gulf Coast	32 (23%)	31 (17%)	63 (19%)
Apalachicola River	28 (20%)	19 (10%)	47 (15%)
Appalachian Mountains	9 (6%)	23 (12.5%)	32 (10%)
Mississippi River	12 (9%)	46 (25%)	58 (18%)
Apalachicola River/Appalachian Mountains and Mississippi River	5 (4%)	3 (2%)	8 (3%)
Florida Peninsula	4 (3%)	36 (20%)	40 (12%)
Laurentide Ice Sheet	1 (0.7%)	17 (9%)	18 (6%)
Other riverine	Not categorized	23 (12.5%)	23 (7%)
Other	39 (28%)	23 (12.5%)	60 (19%)
None	36 (26%)	49 (27%)	85 (26%)
Total studies	140	184	324

Note

Some studies showed more than one discontinuity, such that the number of observations sum to more than the total number of studies listed in Table 2. For more details about the specific studies summarized in this table, see Table S1.

**FIGURE 3:**
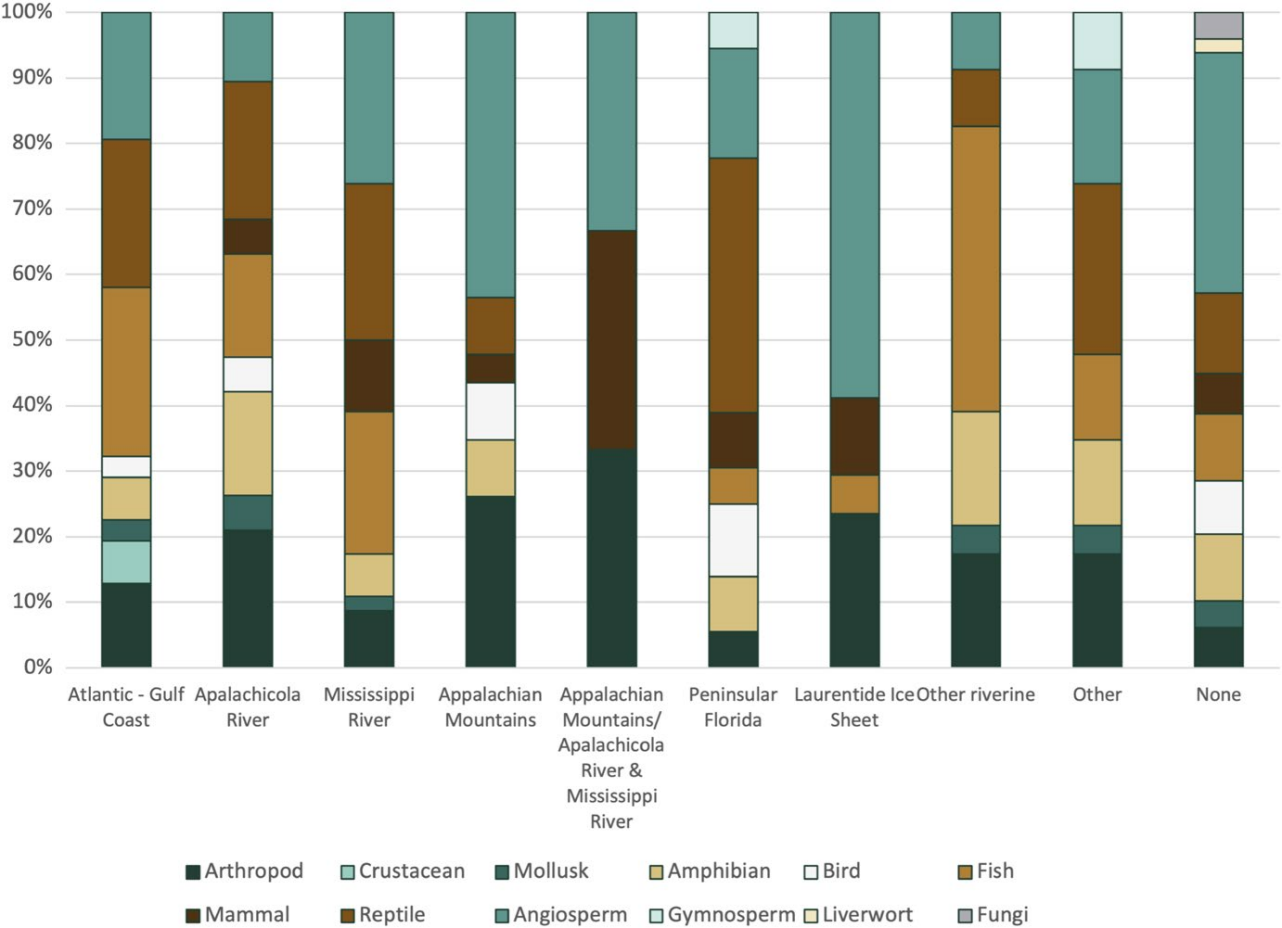
Stacked graphs of the proportion of taxa exhibiting a discontinuity

The Mississippi River and peninsular Florida appear to be important factors shaping discontinuities in reptiles, with 22% (11) and 27% (14) of reptile‐focused studies identifying those discontinuities, respectively (Table S2, Figure 3). The Mississippi River was also an important discontinuity in fish, with 24% (10) of studies identifying this barrier (Table S2, Figure 3). Also important for fish was the Atlantic/Gulf Coast discontinuity (8, or 19%) and Smaller riverine systems (10 or 24%) (Table S2, Figure 3). Although Angiosperm studies most frequently found no pattern (18 or 26%), when a pattern was found it was most often the Mississippi River (12 17%), Appalachian Mountains (10, or 14%) or south of the Laurentide Ice Sheet (10 or 14%) (Table S2, Figure 3). For bird and mammal studies, few common discontinuities were found across taxa, possibly because species vary in vagility, which may lead to variation in the most important factors affecting genetic structure. For most types of invertebrates, fungi, liverworts, and gymnosperms, we were unable to determine whether they exhibited common discontinuities because of the very few studies conducted on these taxa.

### 3.2 Support for discontinuities using divergence time estimation

The studies that dated divergence times found support for each of the discontinuities except for the Apalachicola River/Appalachian Mountains and Mississippi River discontinuities. Divergence dates ranged from mid‐Miocene (Langhian Age, 15.3 [11.8–19.5] mya; Martin et al., 2013) to the mid to late Pleistocene (0.039–0.217 [0.004–0.402] mya; Peterson & Graves, 2016). Around half of these papers (26, or 56.52%) concluded that divergences possibly occurred prior to the Pleistocene (Table S1). For a detailed description of whether divergence time estimation supported the expected dates outlined in Table 1, see the description of each discontinuity in the discussion.

## 4 DISCUSSION

### 4.1 Taxonomic focus of phylographical studies

The first goal of the study was to understand the taxonomic focus of phylogeographical studies and whether it has changed over time. Previous literature search‐based review studies using the term “phylogeography” demonstrated a disparity between animal and plant studies: Avise (2000) found that animal studies comprised at least 70% of the phylogeographic studies, whereas Soltis et al. (2006) found that 75% were animal‐focused and only 21% focused on plants. Notably, Soltis et al. (2006) found a particular bias toward studies focused on vertebrates, particularly fish, despite the fact that vertebrates exhibit much lower species diversity than invertebrates, plants, and fungi. Of the 184 papers assessed for the present study, 95 (52%) focused on vertebrates, 31 (17%) focused on invertebrates, 56 (30%) focused on plants, and 2 (1%) focused on fungi. Relative to the Soltis et al. study, the overall proportion of plant studies increased over time from 21% to 30%, although a large disparity still remains in the number of studies conducted in vertebrates vs. plants that is disproportionate to their species diversity. As noted by Soltis et al. (2006), the smaller number of studies conducted in plants relative to vertebrates may be due to differences in the resolution of genetic markers, as mitochondrial DNA sequences in animals traditionally have provided much greater resolution of phylogeographic patterns than plant chloroplast or mitochondrial DNA sequences (Soltis et al., 2006). As such, phylogeographical studies in vertebrates have traditionally been less time‐consuming and more well resolved than in plants, which may have led to greater numbers of publications. However, the advent of NGS has greatly improved the ability to genotype individuals at a large number of variable markers, opening the way for improved phylogeographical inferences in plants, which may increase the number of plant phylogeographical studies going forward.

Relative to the Soltis et al. study, the proportion of studies focused on invertebrates has remained constant at about 15%–17% and the proportion of fungal studies decreased slightly from 3% to 1%. Considering their high species diversity, studies on both fungi and invertebrates are dramatically under‐represented in phylogeographical studies. This in part may reflect the smaller proportion of scientists focusing on these taxa in the scientific community. The phylogeographic factors that have affected these poorly studied taxa are not well known, and this represents a major gap in our understanding of the phylogeography of eastern North America. An increase in the number of studies focused on these ecologically important taxa would undoubtedly help broaden our overall understanding of the phylogeography of the region.

### 4.2 Use of new technology

The second goal of the study was to evaluate the adoption of technologies that can improve the resolution of phylogeographic studies, namely NGS DNA sequencing technologies, divergence time estimation, and niche modeling to test hypotheses.

#### 4.2.1 Next‐generation sequencing

Next‐generation sequencing has become very cost‐effective and can generate genotypic data for a large number of markers distributed throughout the genome, which may greatly improve the resolution of phylogeographic discontinuities; for example, studies that employed NGS approaches more frequently identified phylogeographic discontinuities than those utilizing markers that provide less genomic coverage (i.e., microsatellites) in our study (Figure 2f). Techniques such as RADseq and GBS are increasingly being used to analyze genetic structure and to reconstruct phylogenies (Andrews et al., 2016; Davey & Blaxter, 2010). However, the most common molecular marker employed in the papers examined for this study was Sanger DNA sequencing of a small number of organellar markers, whereas a relatively small proportion of studies examined in the study (34, or 19%) employed NGS genotyping techniques. However, the proportion of phylogeographic studies utilizing NGS technology has increased in recent years, and it will likely increase in the future.

Comparing studies in which a species was initially genotyped by conventional markers and then re‐analyzed using NGS technology can illustrate how employing NGS techniques can improve resolution. One example is two studies focusing on the Loblolly Pine (*Pinus taeda*), both of which covered the same region of the southeastern United States: one study employed 18 microsatellite markers for genotyping 109 individuals (Al‐Rabab’ah & Williams, 2002), whereas the other utilized 23 microsatellite markers and ~3,000 SNPs to genotype 622 individuals (Eckert et al., 2010). Weak genetic structure was identified using microsatellites in both studies, but the combined analysis of microsatellites and SNPs in Eckert et al. (2010) revealed a Mississippi River discontinuity. The SNP data were able to detect further substructure, indicating three genetically distinct groups, suggesting that the use of NGS data as well as additional taxon sampling could help detect fine‐scale genetic diversity.

On the other hand, because the per‐sample cost of NGS genotyping approaches is often higher than traditional approaches, one possible factor that could lead to a spurious detection of a discontinuity is limited sampling; for example, a pattern of isolation by distance may be misinterpreted as a discontinuity if population sampling is sparse. Careful consideration of population sampling will be necessary to avoid this pitfall in studies employing NGS genotyping approaches.

In summary, although uptake of NGS technologies has been gradual (Figure 2b), we anticipate that their use will continue to increase over time. Because of the improved resolution they provide, the use of NGS genotyping approaches may decrease the effort involved in deciphering phylogeographic patterns and consequently increase both the number of phylogeographic studies and their taxonomic diversity going forward. However, careful sampling and experimental design will be necessary to ensure the accuracy of phylogeographic conclusions.

#### 4.2.2 Divergence time estimation

Similarly, divergence time estimation was employed in only a small proportion of studies even though software has been available for this purpose throughout the period of time covered by the present study. All studies that dated divergence times used phylogeny‐based approaches such as BEAST. Approaches that can date intraspecific divergences, such as IM, ∂a∂I, and DIYABC were used in only a handful of studies to test hypotheses regarding the order of divergences among lineages or the demographic history of populations (which can provide important insights into the historical factors affecting species’ distributions), but they were not used to date divergence times. Because a wide range of historical factors may shape divergences among lineages, having an estimate of when those discontinuities arose is crucial for inferring the forces giving rise to phylogeographic discontinuities. Divergence time estimation is also critical for hypothesis testing, which is illustrated by the finding that half of the studies dating divergence times found that divergences likely occurred prior to the Pleistocene, even though many authors assume that the Pleistocene glacial cycles may have given rise discontinuities in species.

The approaches to date divergence times and infer the demographic history of taxa are complex; they require careful consideration and multiple rounds of analyses to calibrate, set priors, or devise evolutionary scenarios. Furthermore, many analyses may fail to provide definitive answers due to insufficient variation in genetic data. However, we assert that whenever possible, divergence time estimation and demographic modeling should be incorporated into phylogeographic studies because of the powerful insights that they provide into the phylogeographic history of taxa.

#### 4.2.3 Ecological niche modelling

Another factor that we evaluated is whether studies employed ENM, but we found that only a few papers (23, or 12.5%) used this approach. ENM can be useful because it can be used to infer how the past environment shaped a divergence; for example, if the divergence occurred during an era when the species experienced an increase in available niche space, then the divergence may have occurred due to a range expansion (e.g., via long‐distance dispersal to a new habitat), whereas if the divergence occurred during an era in which the suitable habitat decreased, the divergence may have occurred due to a range contraction, resulting in a vicariance in the range of a species. ENM has also been used to identify past glacial refugia (Waltari et al., 2007). One example of how ENM can help infer the phylogeographic history of a group is illustrated in a study of *Delphinium exaltatum* (Mohn et al., 2021), where ENM analyses indicated that a divergence occurred due to postglacial northward range expansion. ENM also helped narrow estimates of the divergence time between the two lineages by showing that one lineage showed a major shift in niche availability over a 500‐year period; this gap in niche space would have required very rapid migration rates for this lineage, suggesting that it likely did not exist prior to the shift in niche availability. We recognize that employing ENMs and other related approaches such as iDDC into phylogeographic studies can be difficult due to their theoretical and computational complexity (see Alvarado and Knowles (2013) for a more thorough review of the strengths and drawbacks of these approaches in phylogeography). However, given the utility of these approaches for understanding how past climate fluctuations shaped the niche availability of a species, we recommend that future studies consider employing ecological niche modeling or related approaches to strengthen the ability to test phylogeographical hypotheses.

### 4.3 Known discontinuities

The final goal was to assess the relative strength of evidence for the biogeographic discontinuities proposed by Soltis et al. (2006), whether there is new evidence for any other additional patterns not found previously by Soltis et al. (2006) and whether specific groups of taxa show common discontinuities. One of the most surprising findings was that a large number of papers surveyed here (49, or 27%) found no discontinuity. Although no geographical pattern or barrier existed in a few species, more frequently the authors indicated that the lack of structure might be due to weakness in the design of the study. For example, several authors indicated that the markers employed were not sufficiently variable (Morris et al., 2008; Strickland et al., 2014) or the sample size was too small to resolve patterns of genetic structure within or among populations (Berendzen et al., 2008; Martin et al., 2013). Attributes of the biology of a species may also prevent the detection of population structure. For example, some studies showed very low genetic diversity, possibly because of past inbreeding or genetic bottlenecks (Makowsky et al., 2009). Other taxa showed signs of hybridization or panmixia that obscured patterns of population structure (Ramaiya et al., 2010; Triplett et al., 2010). Some species also showed no phylogeographic patterns because of high mobility and dispersal that would have obscured any phylogeographic patterns (Hodel et al., 2016; Peterson & Graves, 2016). Despite a range of different causes contributing to a lack of a phylogeographic pattern, almost all studies offered two solutions: (1) wider sampling both within populations and across a wider geographic range (if possible) and (2) more markers or markers that provide greater resolution.

#### 4.3.1 Maritime Atlantic Coast/Gulf Coast

The Maritime Atlantic/Gulf Coast (Figure 1, Table 1) discontinuity is characterized by genetic diversity being structured between the Gulf Coast and Atlantic Coasts (i.e., on the west or east coasts of peninsular Florida), with the discontinuity actually occurring somewhere in south Florida (Figure 1). This discontinuity has been observed in multiple plant and animal studies (Avise & Nelson, 1989; Gurgel et al., 2004; Saunders et al., 1986), but it is generally found primarily in species occupying marine or coastal habitats such as fish and marine invertebrates. This discontinuity is largely attributed to habitat‐related barriers to gene flow, including the subtropical climate, presence of mangrove‐dominated ecosystems, and adverse currents in southern Florida, and possibly river drainages (Germain‐Aubrey et al., 2014; Padhi, 2012). In the present study, this pattern was largely found in marine organisms such as fish and mollusks (Table S2, Figure 3).

The studies that found a discontinuity between the Atlantic and Gulf Coast in Florida generally showed a range of locations and divergence times separating lineages. The location of the phylogeographic break between lineages ranged anywhere from the southern tip of Florida to along the east coast of Florida. Seven papers that observed this discontinuity dated divergence times, but no common divergence time distinguished this discontinuity, with dates ranging from the late‐Miocene 6.43 [0.55–13.59] mya (Germain‐Aubrey et al., 2014) to the late Pleistocene 0.33 [0.26–0.39] mya (Barrow et al., 2017). The dates in the Pleistocene suggest that these divergences may have in part been shaped by glacial cycles that dramatically altered the Florida coastline during the Pleistocene. However, because the dates span a large time period, it appears that many different factors may have led to this discontinuity across species.

#### 4.3.2 Apalachicola River

During the interglacial periods in the Pleistocene and earlier epochs, the Apalachicola River (Figure 1, Table 1) and other southern river drainages (i.e., Chattahoochee River, Tombigbee River) are generally thought to have acted as a barrier that led to vicariances between lineages occurring on either side of the river (Bermingham & Avise, 1986; Edwards et al., 2008; Liu et al., 2006). The melting of glaciers during interglacial periods is thought to have caused the expansion of many southern rivers and associated floodplain forests that may have acted as a geographic barrier limiting dispersal in terrestrial organisms. The Apalachicola River bisects the southeastern region of the United States and most likely formed during the glacial cycles during Pleistocene (2.58–0.0117 mya), ultimately draining into the Gulf of Mexico. Species exhibiting this pattern generally show a genetic divergence between populations on the western and eastern sides of the rivers (Bermingham & Avise, 1986; Edwards et al., 2008; Liu et al., 2006).

Although 19 (10%) studies identified this as a potential discontinuity in their study taxon, this was only half the number found in Soltis et al. (2006), likely because of the addition of the Florida peninsula discontinuity. Twelve of the 19 species exhibiting this discontinuity were vertebrates, but this large number is mainly attributable to the greater number of vertebrate studies. Of the studies that identified this discontinuity, only five dated divergence times, with dates ranging from the Pliocene 4.58 [2.06–7.51] mya (Mila et al., 2017) to the late Pleistocene 0.32 [0.1–0.5] mya (Krysko et al., 2017).

Although a moderate proportion of studies identified this as a potential discontinuity in their study taxon, the Apalachicola River discontinuity was often difficult to distinguish from other discontinuities, such as the Gulf/Atlantic Coast discontinuity; for example, only one study that exhibited this discontinuity did not show evidence for an additional discontinuity. While the Apalachicola River and other southern rivers such as the Tombigbee River have been proposed to be geographic barriers for a variety of species, they could also be the result of other more prominent discontinuities, such as the Gulf/Atlantic Coast and Appalachian Mountain discontinuities. Further, other rivers in this region, such as the Tombigbee River and Chattahoochee River, have also been proposed to have acted as significant barriers, making it difficult in some species to confidently determine which river shaped a phylogeographic discontinuity. In almost all cases, additional resolution could be gathered by more dense population sampling in the region.

#### 4.3.3 Appalachian Mountains

Like the riverine barriers proposed by Soltis et al. (2006), the Appalachian Mountains (Figure 1, Table 1) were also proposed as a geographic barrier separating lineages to the east and west (Soltis et al., 2006). The Appalachian Mountains date back over 480 million years and are the result of multiple cycles of geologic uplift, weathering, and erosion. The last uplift occurred during the mid‐Miocene (Poag & Sevon, 1989), leading to land formations as they are currently known. If this most recent uplift affected phylogeographical patterns, then divergence dates of species showing an Appalachian Mountain discontinuity should date to after the mid‐Miocene (14.2 mya). Indeed, of the studies that uncovered an Appalachian Mountain discontinuity and dated the divergence (6 or 40%), all of them were dated to after the mid‐Miocene. The oldest divergence dates were 8 [7.1–8.3] mya in the four‐toed salamander (*Hemidactylium scutatum)* (Herman & Bouzat, 2016) and 5.4 [3.1–8.1] mya for the Southern Red‐backed Salamander (*Plethodon serratus*) (Thesing et al., 2016). However, many studies have also attributed this discontinuity to species occupying separate glacial refugia east and west of the Appalachians. Indeed, the majority of studies found divergence times dating to the Pliocene and Pleistocene, with the most recent divergence occurring 0.806 (0.454–1.211) mya within the four‐toed salamander (*Hemidactylium scutatum*; Herman & Bouzat, 2016).

Overall, we found more support for this pattern than was previously found in Soltis et al. (2006), with greater support for multiple Pleistocene refugia shaping this discontinuity. However, due to other geographic features that could have affected phylogeographic patterns in the area, such as rivers, it is often challenging to confidently determine whether phylogeographical discontinuities were shaped by the Appalachians. Indeed, this discontinuity was also frequently cited in conjunction with the Apalachicola River discontinuity, as both barriers divide the southeastern United States.

#### 4.3.4 Mississippi River

The Mississippi River (Figure 1, Table 1) may have been a barrier throughout the Pleistocene (2.58–0.0177 mya), at which time its flow, size, and course were greatly altered due to cyclic glaciations (Hobbs, 1950; Leverett, 1921; Soltis et al., 2006), along with the expansion of floodplain forests that may have acted as a barrier to dispersal. Lineages exhibiting this discontinuity are expected to exhibit genetic differentiation and substructure east and west of the Mississippi River, which was observed in many species (e.g., Al‐Rabab'ah & Williams, 2002; Near et al., 2001). Consistent with these expectations, 63% of the papers that found a Mississippi River discontinuity and dated divergences found that the divergence occurred during the Pleistocene, with the most recent divergence event occurring 0.13 [0.06–0.21] mya in striped skunks (*Mephitis mephitis*) (Barton & Wisely, 2012).

The present study found nearly four times as many studies showing a Mississippi River discontinuity than the Soltis et al. (2006) study, providing a great deal more evidence for the importance of this geographic barrier. It appears to have been a particularly strong barrier for reptiles, with 11 reptile taxa showing a Mississippi River discontinuity. It also appears to be an established barrier for fish; as the river receded after the Pleistocene interglacial periods, smaller riverine systems began to form, separating species of fish that were once previously connected by the expansion of the river. Overall, recent work demonstrates the Mississippi River likely has had strong impact on the phylogeographic patterns in many eastern North American taxa and that divergences occurred most frequently during the Pleistocene.

#### 4.3.5 Appalachian Mountains/Apalachicola River and Mississippi River

The Appalachian Mountains/Apalachicola River and Mississippi River (Figure 1, Table 1) discontinuity involves three geographically structured genetic groups: one located west of the Mississippi River, one located between the Mississippi River and Apalachicola River (or Appalachian Mountains), and one located east of the Apalachicola River (or Appalachian Mountains). This pattern again was proposed to be due to the expansion of rivers and the existence of multiple refugia during Pleistocene interglacial periods. Soltis et al. (2006) found this pattern in five studies: two in animals (Brant & Ortí, 2003; Burbrink et al., 2009), one in plants, American bellflower (*Campanulastrum americanum*) (Barnard‐Kubow et al., 2015), and two in arthropods (Hill, 2015; Stephens et al., 2011). In the present study, we found only one study in short‐winged grasshoppers (*Melanoplus scudderi*) that potentially demonstrated this discontinuity (Hill, 2015), but with only weak support for a group east of the Apalachicola River. It is possible that such few studies match to this geographic barrier because few taxa may have had distributions that were affected by both the Mississippi River and the Apalachicola River simultaneously.

#### 4.3.6 Laurentide Ice Sheet

The Laurentide Ice Sheet covered a large part of the northern portion of North America during the last glacial maximum when it extended as far south as approximately 39°N (Figure 1). Species may have occupied one or multiple refugia south of the Laurentide Ice Sheet during the glacial periods, and, when the ice sheet receded, the formerly glaciated areas were recolonized through northward expansions. Until recently, this post‐glacial northward re‐colonization was thought to result in lower genetic diversity in Northern populations due to founder effects or bottlenecks, with greater genetic diversity expected to occur in the south, as was found in Europe (Demesure et al., 1996; Hewitt, 1999; Taberlet et al., 1998; but see Birks & Willis, 2008). However, a recent study by Lumibao et al. (2017) found that many eastern North America plant populations that occur in northern areas close to the Laurentide Ice Sheet limits unexpectedly maintained comparable levels of genetic diversity to those found in more southern populations. This is possibly due to species occupying more refugia in eastern North American, possibly in areas just south of the Laurentide Ice Sheet, in contrast to a very limited number of refugia in the Mediterranean peninsulas in European species. Although these patterns complicate the detection of refugia in North America, employing techniques such as demographic modeling and ecological niche modeling, which are powerful tools to assess phylogeographic patterns, likely may help solve this problem. Although the presence of refugia just south of the Laurentide Ice Sheet was previously acknowledged by Soltis et al. (2006), recent work has highlighted its importance in shaping the geographic distribution of genetic variation in North America.

### 4.4 Support for newly recognized discontinuities

#### 4.4.1 Florida Peninsula

Although recognition of the Florida peninsula as a discontinuity is not novel, our study is the first to consider it within the context of the other discontinuities commonly found in the southeastern United States. For this review, the peninsular Florida discontinuity refers to a line roughly running from around Jacksonville, FL, directly west to the Gulf of Mexico, which separates peninsular Florida from continental North America (Figure 1). This discontinuity involves one or more lineages isolated to the Florida peninsula while others are found just north of the peninsula or elsewhere in the southeast. This differs from other discontinuities previously established in this region; for example, species exhibiting the Atlantic/Gulf discontinuity have genetic groups structured according to the coast they occupy, whereas species exhibiting the Florida peninsular discontinuity have at least one lineage distributed throughout the peninsula, usually centrally located. It is also possible for this discontinuity to occur in conjunction with others in the region.

The Florida peninsula is only one small part of a larger Florida platform, most of which is currently underwater. The formation of the Florida peninsula as we know it occurred in the Miocene when the entire peninsula was submerged, with marine deposits forming a portion of the higher‐elevation regions of Florida (Germain‐Aubrey et al., 2014; Webb, 1990). Throughout the Pleistocene, the unsubmerged areas of the Florida platform varied depending on the glacial cycles. At the peak of the last glacial maximum, the entire Florida platform was exposed. During interglacial periods, only a small portion of peninsular Florida was above water and these higher‐elevation areas formed an archipelago, which acted as a barrier to gene flow and is the most likely factor underlying this discontinuity. As the glacial period came to an end, more of the platform was submerged, forming the present‐day Florida coastline.

Overall, we identified 36 (20%) studies that found a Florida peninsular discontinuity. Over a third (14, or 39%) of the papers studying reptiles found a peninsular Florida discontinuity. Just over a third of the studies (13, or 36%) also dated divergence times, ranging from the mid‐Miocene (6.64 mya) to the late Pleistocene (0.4 mya) with no epoch favored. This indicates that divergences may have occurred at any time throughout the latter half of the Cenozoic Era due to frequent fluctuations in sea level. During the Miocene and Pliocene when nearly the entire Florida Platform was underwater except the highest elevation ridges in the center of the peninsula, many species likely diverged because they were isolated to those central ridges. For example, gopher frogs, *Lithobates capito* (Richter et al., 2014) show two peninsular lineages that diverged from the coastal plain lineage 2.3 [1.4–3.1] mya during the late Pliocene and early Pleistocene when the ridges were still archipelagos. The oak species *Quercus geminata* and *Q*. *minima* are range restricted to peninsular Florida and diverged from their widespread southeastern sister species *Q*. *virginiana* in the Miocene around 8 mya (Cavender‐Bares et al., 2015). Two studies in the lizard *Anolis carolinensis* demonstrated that the species spread from south to north within peninsular Florida during the Miocene and Pleistocene (Campbell‐Staton et al., 2012; Manthey et al., 2016). Soltis et al. (2006) observed four papers with a peninsular Florida pattern of discontinuity but did not discuss this discontinuity. Overall, however, the present study demonstrates that this discontinuity could be a significant factor affecting the phylogeography of species in southeastern North America.

## 5 CONCLUSIONS AND FUTURE DIRECTIONS

Although the comparative phylogeography of taxa in eastern North America has been assessed frequently throughout the last 30 years, much is still unknown about the factors affecting the geographic patterns of genetic variation of the species in the region. Relatively few phylogeographical studies in the region have utilized new technologies such as NGS genotyping approaches that improve genetic resolution. Also lacking is the uptake of analytical approaches that improve the ability to test phylogeographical hypotheses, such as divergence time estimation, demographic modeling, and ecological niche modeling. Thus, the resolution of many studies has been limited, which has limited our ability to assess the strength of support for phylogeographical discontinuities. However, detection of the factors shaping patterns of genetic variation between species or populations is crucial for understanding and conserving biodiversity; with increasing threats of climate change, continued land development, and rapidly decreasing habitat availability, accurately understanding the past geographic and climatological factors that have shaped genetic diversity and structure is essential for understanding species’ responses to future stressors. Thus, it is imperative that future studies begin to employ technologies and analytical approaches that will improve their ability to test the phylogeographical hypotheses and discontinuities in eastern North America discussed both in Soltis et al. (2006) and in the present study. We also highlight the need for improved taxonomic diversity in phylogeographical studies to broaden our inference of the generality of the forces shaping the geographical patterns of genetic variation across species. By improving the taxonomic diversity and implementing more rigorous hypothesis testing, we will certainly improve our knowledge of how these species came to be and how that knowledge may benefit the long‐term survival of biodiversity.

## AUTHOR CONTRIBUTIONS

**Rachel Ann Lyman:** Conceptualization (equal); Data curation (lead); Formal analysis (lead); Methodology (equal); Visualization (lead); Writing – original draft (lead); Writing – review & editing (equal). **Christine E. Edwards:** Conceptualization (equal); Methodology (equal); Supervision (lead); Writing – original draft (supporting); Writing – review & editing (equal).

## Supporting information

Supplementary Material

## Data Availability

These data were derived from publicly available publications accessed through Web of Science. The main information used as the basis for the analysis is provided in Table S1 and citations are provided in Appendix S1.
