# Supplementary material for: Revisiting the comparative phylogeography of unglaciated eastern North America: 15 years of patterns and progress
Source: Ecol Evol. 2022 Apr 19;12(4):e8827. doi: 10.1002/ece3.8827 (PMC9019306; doi:10.1002/ece3.8827)
Supplement: Supplementary file 1 — Supplementary Material [file ECE3-12-e8827-s001.docx]

**Supporting Information for:**

Revisiting the comparative phylogeography of unglaciated eastern North America: 15 years of patterns and progress

Rachel Ann Lyman and Christine E. Edwards

**Table of Contents:**

| **Table S1** | Page 2-22 |
| --- | --- |
| **Table S2** | Page 23 |
| **Appendix S1** | Page 24-43 |

Table S1. Information about the papers examined in the study. The table includes the taxa, hierarchical taxonomic level, type of molecular marker, phylogeographic pattern revealed in the study, divergence date (if examined in the study, and citation for the study. All references are provided in Appendix S1.

| **TAXON** | **INTER-/INTRA- SPECIFIC** | **MARKERS** | **PATTERN** | **DIVERGENCE DATE** | **REFERENCE** |
| --- | --- | --- | --- | --- | --- |
| **FUNGI** |  |  |  |  |  |
| Caesar Mushrooms  (11 Amanita species) | Interspecific | nu DNA seq | None - cryptic species | None | Sanchez-Ramirez et al., 2015 |
| Madagascar Parmotrema Lichen  (Parmotrema madagascariaceum)  Madagascar Ruffle Lichen  (Parmotrema xanthinum) | Intraspecific | nu DNA seq | None - needs more molecular work and biogeographic history | None | Lendemer, 2016 |
| **PLANT (ANGIOSPERM)** |  |  |  |  |  |
| Sugar Maple  (Acer saccharum) | Intraspecific | microsatellites; cp DNA seq | Appalachian Mountains (E pp = ?; W pp= 0.97); Other | Species split 11.5 [6.6-16.7] mya, Miocene; lineage diversification 5.4-1.4 [0.2-7.3] mya late Miocene-Pleistocene | Vargas-Rodriguez et al., 2015 |
| Red Maple  (Acer rubrum)  Silver Maple  (Acer saccharinum) | Intraspecific | cp DNA seq | Florida Peninsula (A. rubrum)  Appalachian Mountains; Laurentide Ice Sheet (A. saccharinum) | None | Saeki et al., 2011 |
| Common Ragweed  (Ambrosia artemisiifolia) | Intraspecific | GBS (SNPs) | Appalachian Mountains | None | Martin et al., 2016 |
| Hill Cane  (Arundinaria appalachiana)  Giant Cane  (Arundinaria gigantea)  Switchcane  (Arundinaria tecta) | Interspecific | AFLPs; cp DNA seq | None - hybridization; distinct morphology; greater sampling | None | Triplett et al., 2010 |
| Burks Smooth Rockcress  (Boechera burkii)  Sicklepod Rockcress  (Boechera canadensis)  Smooth Rockcress  (Boechera laevigata)  Missouri Rockcress  (Boechera missouriensis)  Braun's Rockcress  (Boechera perstellata)  Shale Barren Rockcress  (Boechera serotina)  Short's Rockcress  (Boechera shortii) | Interspecific | cp DNA seq | Laurentide Ice Sheet | None | Kiefer et al., 2009 |
| Tall Bellflower  (Campanulastrum americanum) | Intraspecific | cp DNA seq; RAD-seq (SNPs) | Mississippi River (pp=1; Bootstrap = 98) (pp=0.95); Appalachian Mountains (pp = .7; Bootstrap = <50) (pp=0.74) and (?) (pp= 0.64) | Appalachian clade: 2.3–7.0 mya, the Eastern and Western clade: 0.7–2.3 mya; | Barnard-Kubow et al., 2015 |
| Broom Sedge  (Carex scoparia)  Broom Sedge  (Carex waponahkikensis) | Interspecific | microsatellites | None - need more extensive sampling through outside of Maine | None | Escudero et al., 2019 |
| Bitternut Hickory  (Carya cordiformis)  Shagbark Hickory  (Carya ovata) | Intraspecific | ddRADseq | Laurentide Ice Sheet | None | Bemmels and Dick, 2018 |
| American Chestnut  (Castanea dentata)  Ozark Chinquapin  (Castanea ozarkensis;  Castanea pumila var. ozarkensis)  Allegheny Chinquapin  (Castanea pumila;  Castanea pumila var. pumila) | Interspecific | cp DNA seq | Other | None | Shaw et al., 2012 |
|  | Both | cp and nu DNA seq | None- hybrid zone; further gene resolution | None | Li, Xiaowei; Dane, Fenny, 2013 |
| Sand-heath  (Ceratiola ericoides) | Intraspecific | allozymes | Florida Peninsula; Other | None | Trapnell et al., 2007 |
| American Cancer-root  (Conopholis americana) | Intraspecific | microsatellites | Laurentide Ice Sheet | None | Rodrigues et al., 2016 |
| Flowering Dogwood  (Cornus florida) | Intraspecific | microsatellites | None- high dispersal; large Ne | None | Hadziabdic et al., 2010 |
| Balms  (11 Dicerandra species) | Interspecific | nu and plastid DNA seq | Atlantic-Gulf (Bootstrap = 99); Florida peninsula (Bootstrap 100) | None | Oliveira et al., 2007 |
| Eastern Leatherwood  (Dirca palustris) | Both | cp DNA region seq | None- IBD, long distance dispersal; limited evidence of contemporary dispersal; Laurentide Ice Sheet | Species split 0.623 [0.247-1.1] mya, Pleistocene; within lineage diversification 0.039 -0.217 [0.004-0.402] mya (Pleistocene) | Peterson and Graves, 2016 |
| American Beech  (Fagus grandifolia) | Intraspecific | cp DNA seq | None- greater resolution; need nu DNA seq; expand sampling | None | Morris et al., 2010 |
| Ivyleaf Morning-glory  (Ipomoea hederacea) | Intraspecific | nu DNA seq; Sanger | None - need to look at cp DNA; long distance dispersal; IBD | None | Campitelli and Stinchcombe, 2014 |
| Lamance Iris  (Iris brevicaulis)  Red Iris  (Iris fulva) | Both | GBS (SNPs) | None- low pop structure; asymmetric hybridization; IBD | None | Hamlin and Arnold, 2014 |
| Dixie Iris  (Iris hexagona) | Intraspecific | GBS (SNPs) | Apalachicola River | None | Hamlin and Arnold, 2015 |
| Panhandle lily  (Lilium iridollae)  Carolina lily  (Lilium michauxii)  Sandhills Lily  (Lilium pyrophilum)  Turk’s cap lily  (Lilium superbum) | Interspecific | cp and nu DNA seq | None- asymmetrical gene flow | 0.188 [0.084-0.586] mya | Douglas et al., 2011 |
| Pondberry  (Lindera melissifolia) | Intraspecific | microsatellites | Appalachian Mountains | None | Echt et al., 2011 |
| Sweetgum  (Liquidambar styraciflua) | Intraspecific | cp DNA seq | None - high dispersal distance; cp DNA slow evolution; need new markers | Species split 14.44 [6.51-31.64] mya | Morris et al., 2008 |
| Tuliptree  (Liriodendron tulipifera) | Intraspecific | cp and nu DNA seq | Atlantic-Gulf, Apalachicola and Tombigbee Rivers, Appalachian Mountains, Mississippi, Mississippi and Apalachicola River, Peninsular Florida | None | Fetter and Weakley, 2019 |
| Water Tupelo  (Nyssa aquatica)  Swamp Blackgum  (Nyssa biflora)  Ogeechee Tupelo  (Nyssa ogeche)  Blackgum  (Nyssa sylvatica)  Dwarf Blackgum  (Nyssa ursina) | Interspecific | ddRADseq; GBS | None - more extensive sampling of N. biflora and N. ursina | None | Zhou et al., 2018 |
| Prickly-pear  (Opuntia abjecta)  Eastern Prickly-pear  (Opuntia humifusa)  Twisted-spine Prickly-pear  (Opuntia macrorhiza)  Little Prickly-pear  (Opuntia pusilla)  Prickly-pear  (Opuntia tortispina)  Twisted-spine Prickly-pear  (Opuntia xanthoglochia) | Interspecific | nu DNA seq | None- unclear spp boundaries; polyploidy | None | Majure et al., 2012 |
| Old Switch Panicgrass  (Panicum virgatum) | Intraspecific | SNPs GBS | Laurentide Ice Sheet | None | Grabowski et al., 2014 |
| Slash Pine  (Pinus elliottii)  Loblolly Pine  (Pinus taeda) | Intraspecific | exome sequencing | None (P. elliottii)  Mississippi River (P. taeda) | None | Acosta et al., 2019 |
| Loblolly Pine  (Pinus taeda) | Intraspecific | Illumina (SNPs); microsatellites | Mississippi River | None | Eckert et al., 2010 |
| Hornleaf Riverweed  (Podostemum ceratophyllum) | Intraspecific | cp DNA seq | Laurentide Ice Sheet | None | Fehrmann et al., 2012 |
| Eastern Cottonwood  (Populus deltoides) | Intraspecific | SNPs from targeted sequencing of genes and intergenic regions | Appalachian Mountains | None | Fahrenkrog et al., 2017 |
| Kudzu  (Pueraria montana var. lobata) | Intraspecific | microsatellites; cp DNA seq | None - low mobility and expansion; highly clonal | None | Bentley and Mauricio, 2016 |
| Plateau Live Oak  (Quercus fusiformis)  Sand Live Oak  (Quercus geminata)  Dwarf Live Oak  (Quercus minima)  Live Oak  (Quercus virginiana) | Interspecific | RADseq (SNPs); microsatellites; cp DNA seq; | Florida Peninsula | 9.3 [6.9–11.7] mya southeastern clade; no date specified for Peninsular species | Cavender-Bares et al., 2015 |
| Rose Gentians  (17 Sabatia species) | Interspecific | cp DNA seq | Mississippi River (pp=1, MP Bootstrap = 100, ML Bootstrap = 100); Atlantic-Gulf (pp = 0.93, MP Bootstrap =<50, ML Bootstrap = 64) | Mississippi River: mid- to late Miocene 8.87 [5.72 - 11.98] mya; Atlantic-Gulf: late Miocene 4.34 [2.09-6.78] mya | Mathews et al., 2015 |
| Pitcherplants  (18 Sarracenia species) | Interspecific | nu and cp DNA seq | Atlantic-Gulf | None | Stephens et al., 2015 |
| Trumpet Pitcherplant  (Sarracenia alata) | Intraspecific | microsatellites; cp DNA seq | Mississippi River (P <= 0.001, Bootstrap = ?) | None | Koopman and Carstens, 2010 |
|  | Intraspecific | mt and nu DNA seq; Sanger | Mississippi River (pp=1) | None | Carstens and Satler, 2013 |
|  | Intraspecific | cp and nu DNA; mt DNA gene seq | Mississippi River (pp=?); Other Riverine | None | Satler and Carstens, 2016 |
|  | Intraspecific | SNPs | Mississippi River | None | Zellmer et al., 2012 |
|  | Intraspecific | SNP (ddRAD) | Mississippi River | None | Satler and Carstens, 2017 |
| Biltmore's Greenbrier  (Smilax biltmoreana)  Upright Greenbrier  (Smilax ecirrhata)  Smooth Herbaceous Greenbrier  (Smilax herbacea)  Huger's Carrion-flower  (Smilax hugeri)  Herbaceous Greenbrier  (Smilax lasioneuron)  Downy Carrion-flower  (Smilax pulverulenta) | Interspecific | AFLPs; cp DNA seq | Appalachian Mountains (Bootstrap <=70) | Pleistocene 1.09 [0.58-1.68] mya | Li et al., 2013 |
| Tall Goldenrod  (Solidago altissima) | Intraspecific | microsatellites; cp DNA seq | Mississippi River; Laurentide Ice Sheet | clade to 0.7–2.3 mya, and the divergence of the Western clade | Sakata et al., 2015 |
| Saltwater Cordgrass  (Spartina alterniflora) | Intraspecific | cp DNA seq; microsatellites | Other | None | Blum et al., 2007 |
| American Basswood  (Tilia americana) | Intraspecific | cp DNA seq | None - little structure; low rates of gee flow; possible refugia | None |  |
| Little Sweet Trillium  (Trillium cuneatum) | Intraspecific | cp DNA seq | Appalachian Mountains | None | Gonzales et al., 2008 |
| Little Sweet Trillium  (Trillium cuneatum)  Blue Ridge Trillium  (Trillium stamineum) | Intraspecific | cp DNA seq | Other Riverine | None | Wallace and Doffitt, 2013 |
| Sea Oats  (Uniola paniculata) | Intraspecific | cp DNA region seq | Atlantic-Gulf | None | Hodel and Gonzales, 2013 |
| Hobblebush  (Viburnum lantanoides) | Intraspecific | RADseq | Laurentide Ice Sheet (Bootstrap>90) | None | Park. And Donoghue, 2019 |
| Possumhaw  (Viburnum nudum) | Intraspecific | RADseq | None - possible other boundaries (ie habitat) | did date divergence times, but no clear pattern | Spriggs et al., 2019 |
| Small-leaf Viburnum  (Viburnum obovatum)  Smooth Blackhaw  (Viburnum prunifolium)  Rusty Blackhaw  (Viburnum rufidulum) | Both | RADseq | Laurentide Ice Sheet BS=100 (V.lentago)    Appalachian Mountains BS=100 (V. prunifolium)    Mississippi River BS= 100 (V. rufidulum) | None | Spriggs et al., 2019 |
| Black Mangroves  (Avicennia germinans)  Red Mangroves  (Rhizophora mangle) | Intraspecific | microsatellites | None- high dispersal capability; high genetic connectivity; little structure; use diffferent markers | None | Hodel et al., 2016 |
| Red Mangroves  (Rhizophora mangle) | Intraspecific | microsatellites | Atlantic-Gulf | None | Kennedy et al., 2017 |
|  | Intraspecific | microsatellites; ddRAD-seq | Atlantic-Gulf | None | Hodel et al., 2017 |
| American Holly  (Ilex opaca var. arenicola)  Scrub Bay  (Persea humilis)  Lewton's Polygala Milkwort  (Polygala lewtonii)  Scrub Plum  (Prunus geniculata) | Intraspecific | nu and plastid DNA seq | None (I. opaca)    Florida Peninsula  BS= 100 (P. humilis)  BS=100 (P. lewtonii)  BS=99 (P. geniculata) | Prunus: 2.68 [0.62-5.17] mya Pliocene; Polygala: 6.43 [0.55-13.59] mya Miocene; Persea: 2.38 [0.65-4.78] mya Pleistocene; Ilex: 1.45 [0.38-2.87] mya. Pleistocene | Germain-Aubrey et al., 2014 |
| **PLANT (GYMNOSPERM)** |  |  |  |  |  |
| Bald Cypress  (Taxodium distichum var. distichum)  Pond Cypress  (Taxodium distichum var. imbricarium) | Intraspecific | Sanger; NGS targeted amplicon sequencing (TAS) approach | Florida Peninsula; Other: Texas, Mississippi River Alluvial Valley | None | Ikezaki et al., 2016 |
|  | Intraspecific | microsatellites | Florida Peninsula; Other: Mississippi River Valley | None | Tanaka et al., 2012 |
| **PLANT (LIVERWORT)** |  |  |  |  |  |
| Asa Gray's Scalewort  (Frullania asagrayana) | Intraspecific | microsatellites; plastid and nu DNA seq | None - panmixia; clonal; reproductive isolation | None | Ramaiya et al., 2010 |
| **INVERTEBRATE (ARTHROPOD)** |  |  |  |  |  |
| Central Stonefly  (Acroneuria frisoni) | Intraspecific | mt DNA seq | Mississippi River | None | Pessino et al., 2014 |
| Cactus Moth  (Cactoblastis cactorum) | Intraspecific | mt DNA seq | Atlantic-Gulf | None | Simonsen et al., 2008 |
| Southern Pine Beetle  (Dendroctonus frontalis) | Intraspecific | microsatellites | Apalachicola River; Appalachian Mountains | None | Schrey et al., 2011 |
| American Dog Tick  (Dermacentor variabilis) | Intraspecific | Mt DNA seq | None - more/better molecular markers | None | Kaufman et al., 2018 |
| Pitcher Plant Moth  (Exyra semicrocea) | Intraspecific | mt DNA seq | Mississippi River Atlantic-Gulf; Apalachicola River; Other Riverine | None | Stephens et al., 2011 |
| Southeastern Field Cricket  (Gryllus rubens)  Texas Field Cricket  (Gryllus texensis) | Both | mt DNA seq | Other | Pleistocene 0.25-2 mya | Gray et al., 2008 |
| Buckmoths  (8 Hemileuca spp.) | Interspecific | mt DNA and nuclear genes | None - more extensive sampling needed | None | Rubinoff et al., 2017 |
| Forest Tent Caterpillar Moth  (Malacosoma disstria)  Eastern Tent Caterpillar Moth  (Malacosoma americana) | Intraspecific | Mt DNA seq | Laurentide Ice Sheet | None | Lait and Hebert, 2018 |
| Scudder's Short-winged Grasshopper  (Melanoplus scudderi) | Interspecific | mt DNA seq | Mississippi River; Maybe Mississippi/Apalachicola Rivers: W Mississippi River (Bootstrap = 76) - E Mississippi/W Apalachicola River (Bootstrap = 97) - E Apalachicola River (Bootstrap = 46) | None | Hill, 2015 |
| Eastern Cactus-boring Moth  (Melitara prodenialis) | Intraspecific | mt and nu DNA seq | Florida peninsula; Other riverine | None | Marsico et al., 2015 |
| Worm Millipede  (Narceus americanus)  Worm Millipede  (Narceus annularis)  Smokey Oak Millipede  (Narceus gordanus) | Interspecific | mt DNA seq | Appalachian Mountains (pp = 0.95, bootstrap = 95); Other | None | Walker et al., 2009 |
| Black-Nosed Conehead  (Neoconocephalus melanorhinus) | Intraspecific | AFLPs | Atlantic-Gulf; Other (North Atlantic) | None | Ney and Schul, 2017 |
| Redheaded Pine Sawfly  (Neodiprion lecontei) | Intraspecific | ddRADseq (SNPs) | Other; Laurentide Ice Sheet | Pleistocene (south split from North/Central) ~45kya | Bagley et al., 2017 |
| North American Firefly  (Photinus pyralis) | Intraspecific | ddRADseq; luciferase and opsin genes | Appalachia Mountains | None | Lower et al., 2018 |
| Eastern Subterranean Termite (Reticulitermes flavipes) | Intraspecific | mt and nu DNA seq | Laurentide Ice Sheet | Northern cluster divergence = ABC:64.8 [26.4-115] kya; BEAST: 131.9 [83.6-195] kya | Hyseni and Garrick, 2019 |
| Light Southeastern Subterranean Termite (Reticulitermes hageni)  Eastern Subterranean Termite (Reticulitermes flavipes)  Dark Southern Subterranean Termite (Reticulitermes virginicus) | Intraspecific | mt DNA seq | Florida Peninsula | None | Szalanski et al., 2008 |
| Cavernicolous Harvestman  (Sabacon cavicolens) | Intraspecific | mt and nu DNA seq; Sanger | Other riverine; Ozarks and Appalachian Mountains | None | Hedin et al., 2017 |
| Fall Armyworm Moth  (Spodoptera frugiperda) | Intraspecific | mt DNA seq | Appalachian Mountains | None | Nagoshi et al., 2012 |
|  | Intraspecific | mt DNA seq | Apalachicola River; Other riverine | None | Nagoshi et al., 2014 |
| Mites  (37 Torrenticola species) | Interspecific | mt and r DNA seq | Other | None | Fisher et al., 2017 |
| Fungus Gardening Ant  (Trachymyrmex septentrionalis) | Intraspecific | mt DNA seq | None - more complex; greater mol. Resolution; possible Mississippi River | None | Seal et al., 2015 |
| Pitcher-plant Mosquito  (Wyeomyia smithii) | Intraspecific | RADseq (SNPs) | Atlantic-Gulf; Laurentide Ice Sheet | None | Merz et al., 2013 |
| Eastern Carpenter Bee  (Xylocopa virginica) | Intraspecific | microsatellites | Appalachian Mountains (AMOVA P < 0.00001) | None | Vickruck et al., 2017 |
| **INVERTEBRATE (CRUSTACEAN)** |  |  |  |  |  |
| Florida grass shrimp  (Palaemon floridanus) | Intraspecific | mt DNA seq | Atlantic-Gulf (pp=1) | None | Baeza et al., 2013 |
| Peppermint Shrimp  (Lysmata wurdemanni) | Intraspecific | mt DNA sequence | Atlantic-Gulf | None | Baeza, and Prakash, 2019 |
| **INVERTEBRATE (MOLLUSC)** |  |  |  |  |  |
| Western Fanshell  (Cyprogenia aberti)  Fanshell  (Cyprogenia stegaria) | Interspecific | mt DNA seq; microsatellites | Mississippi River (pp=0.69) | None | Chong et al., 2016 |
| Spectaclecase  (Cumberlandia monodonta) | Intraspecific | mt DNA seq; microsatellites | None- pop fragmentation; more sampling; finer resolution | None | Inoue et al., 2014 |
| Snuffbox mussel  (Epioblasma triquetra) | Intraspecific | microsatellites; mt DNA seq | Other riverine | None | Zanatta et al., 2008 |
| Hooked Mussel  (Ischadium recurvum) | Intraspecific | mt DNA seq | Atlantic-Gulf, Carolinas | None | Fontanella et al., 2019 |
| Conch  (Melongena bicolor)  Florida Crown Conch  (3 Melongena corona ssp.)  Conch  (Melongena sprucecreekensis) | Interspecific | mt DNA seq; r DNA seq | None - no diversity; need use of models | None | Hayes and Karl, 2009 |
| Washboard  (Megalonaias nervosa)  Washboard  (Megalonaias nickliniana) | Both | mt and nu DNA seq | Apalachicola and associated Rivers PP=94 | None | Pfeiffer et al., 2018 |
| **VERTEBRATE (AMPHIBIAN)** |  |  |  |  |  |
| Reticulated Flatwoods Salamander  (Ambystoma bishopi)  Frosted Flatwoods Salamander  (Ambystoma cingulatum) | Interspecific | mt DNA seq | Apalachicola River (1); Other Riverine: Suwannee? (pp=.93, ml bootstrap=77; mp bootstrap=100 | None | Pauly et al., 2012 |
| Dusky Salamanders  (15 Desmognathus species) | Interspecific | mt DNA seq | Atlantic-Gulf | None | Beamer and Lamb, 2008 |
| Eastern Narrow-mouthed Toad (Gastrophryne carolinensis) | Intraspecific | mt DNA seq; AFLPs | None - pop bottleneck and range expansion; sample more related species | None | Makowsky et al., 2009 |
|  |  |  |  |  |  |
| Spring Salamander  (Gyrinophilus porphyriticus) | Intraspecific | mt and nu DNA seq | Other (historical drainages) | Miocene-Pliocene 9.3-4.9 [3.5-13.4] mya for spp complex | Kuchta et al., 2016 |
| Four-toed Salamander  (Hemidactylium scutatum) | Intraspecific | mt DNA gene seq | Appalachian Mountains (pp=0.91, Bootstrap = 89); Other riverine | Whole complex 8 mya (Miocene); within lineage diversification 0.15 mya (Pleistocene) | Herman and Bouzat, 2016 |
| Gopher Frog  (Lithobates capito) | Intraspecific | mt DNA seq | Florida peninsula (pp=1) | 22.3 [1.4-31] mya | Richter et al., 2014 |
| Black Warrior Waterdog  (Necturus alabamensis)  Western Waterdog  (Necturus beyeri)  Neuse River Waterdog  (Necturus lewisi)  Common Mudpuppy  (Necturus maculosus)  Dwarf Waterdog  (Necturus punctatus) | Interspecific | Mt DNA sequence | Apalachicola and associated Rivers PP>92; Mississippi River PP>92 | None | Chabarria et al., 2018 |
| Southern Red-backed Salamander (Plethodon serratus) | Intraspecific | mt and nu DNA seq | None- high phenotypic plasticity; more genetic resolution more sampling | None | Newman and Austin, 2015 |
|  | Intraspecific | mt DNA seq | Mississippi River (pp=1, happened twice); Other riverine | Species 5.4 mya Miocene; within lineage diversification 1.9-0.6 mya, Pleistocene | Thesing et al., 2016 |
| Ornate Chorus Frog  (Pseudacris ornata) | Intraspecific | mt DNA; rRNA; microsatellites | None- habitat fragmentation; need finer gene resolution | None | Degner et al., 2010 |
| Red Salamander  (Pseudotriton ruber) | Intraspecific | mt and nu DNA seq | Other (Coastal Plains) | 3.6 [2.8-4.6] mya Pliocene (species split); subspecies 1.4-1.9 mya | Folt et al., 2016 |
| American Bullfrog  (Rana catesbeiana)  Green Frog  (Rana clamitans) | Interspecific | mt DNA seq | Other | None | Austin and Zamudio, 2008 |
| Southern Leopard Frog  (Rana sphenocephala) | Intraspecific | mt DNA; microsatellites | Apalachicola River and Appalachian Mountains (1, 100, 100); Mississippi River 1, 89, 93); Florida Peninsula (1, <= 50, 78); Other Riverine (pp, mp bootstrap, ml bootstrap) | None | Newman et al., 2011 |
| Lesser Siren  (Siren intermedia)  Greater Siren  (Siren lacertina)  Reticulated Siren  (Siren reticulata) | Both | 5 gene sequences | None - more extensive sampling needed | None | Graham et al., 2018 |
| Southern Toad  (Anaxyrus terrestris)  Green Treefrog  (Hyla cinerea)  Squirrel Treefrog  (Hyla squirella)  Southern Leopard Frog  (Rana sphenocephala) | Intraspecific | sequenced nuclear loci; sequenced mt DNA | A. terrestris None    H. cinerea Atlantic-Gulf 1.0    H. squirella Atlantic-Gulf 1.0    R. sphenocephala Peninsular Florida 1.0 | A. terrestris None  H. cinerea 0.33 [0.26-0.39] mya  H. squirella 0.57 [0.46-0.69] mya ;  R. sphenocephala 1.02 [0.87-1.69] mya | Barrow et al., 2017 |
|  |  |  |  |  |  |
| **VERTEBRATE (BIRD)** |  |  |  |  |  |
| Mottled Duck  (Anas fulvigula)  Mallard  (Anas platyrhynchos) | Interspecific | mt and nu DNA seq | Florida peninsula | 0.39 [0.23-0.6] mya Pleistocene | Lavretsky et al., 2014 |
| Red-shouldered Hawk  (Buteo lineatus) | Intraspecific | mt and nu DNA sequences | Peninsular FL | None | Barrowclough et al., 2019 |
| Northern Bobwhite  (Colinus virginianus) | Intraspecific | mt DNA seq | Florida Peninsula; Appalachian Mountains | None | Eo et al., 2010 |
|  | Intraspecific | mt DNA seq | None | Pliocene, Pleistocene | Williford et al., 2016 |
| Yellow-throated Warbler  (Dendroica dominica) | Intraspecific | Mt DNA seq | None - shallow phylogenetic tree | None | McKay et al., 2009 |
| Red-bellied Woodpecker  (Melanerpes carolinus) | Intraspecific | mt and nu DNA seq | Peninsular Florida | None | Barrowclough et al., 2018 |
| Bachman's Sparrow  (Peucaea aestivalis) | Intraspecific | mt and nu DNA seq; microsatellites; Sanger | None- low genetic structure; high genetic variation; panmixia | None | Cerame et al., 2014 |
| Prothonotary Warbler  (Protonotaria citrea) | Intraspecific | ddRADseq | Appalachian Mountains | None | DeSaix et al., 2019 |
| Cerulean Warbler  (Setophaga cerulea) | Intraspecific | microsatellites; mt DNA seq | None- no genetic variability; high dispersal/mobility; anthropogenic disturbance | None | Deane et al., 2013 |
| Barred Owl  (Strix varia) | Intraspecific | mt and nu DNA seq | Apalachicola River; Gulf - Atlantic(Bootstrap 100) | 100-200kya late Pleistocene (a-g) | Barrowclough et al., 2011 |
| **VERTEBRATE (FISH)** |  |  |  |  |  |
| Yellow Bullhead  (Ameiurus natalis) | Intraspecific | mt DNA seq | Atlantic-Gulf; Florida peninsula | None | Padhi, 2013 |
| White Catfish  (Ameiurus catus) | Intraspecific | mt DNA seq | Atlantic-Gulf (pp=1) | 0.71 [0.37–1.1] mya (Pleistocene) | Padhi, 2012 |
| Bluefin Stoneroller  (Campostoma pauciradii)  Ohio Stoneroller  (Campostoma anomalum)  Largescale Stoneroller  (Campostoma oligolepis) | Interspecific | mt DNA seq | None - more sampling; species boundaries in question | None | Blum et al., 2008 |
| Red Shiner  (Cyprinella lutrensis)  Darters  (10 Etheostoma species) | Intraspecific | mt DNA seq | Mississippi River (AMOVA p<0.001) | None | Glotzbecker et al., 2016 |
|  | Interspecific | mt and nu DNA seq | Other | None | Bossu and Near, 2009 |
|  | Interspecific | nu DNA seq | Mississippi River (pp = 0.95) | Pliocene 4.5 [3.3-5.9] mya | Bossu et al., 2013 |
| Tuckasegee Darter  (Etheostoma proeliare) | Intraspecific | mt DNA seq | Mississippi River; Other riverine | None | Lang and Echelle, 2011 |
| Banded Darter  (Etheostoma zonale)  Brighteye Darter  (Etheostoma lynceum) | Interspecific | mt and nu DNA seq | Mississippi River (100/86); Other riverine | Species split 3.7 to 2.5 mya Pliocene; cryptic species 5.3 to 2.7 mya Pliocene; | Halas and Simons, 2014 |
| Greenside Darter  (Etheostoma blennioides) | Intraspecific | mt DNA seq | Other riverine | None | Piller et al., 2008 |
| Topminnow and Killfish  (31 Fundulus species) | Interspecific | mt and nu DNA seq | Atlantic-Gulf; Florida Peninsula; Other Riverine | None | Whitehead et al., 2010 |
| Northern Studfish  (Fundulus catenatus)  Stippled Studfish  (Fundulus bifax)  Southern Studfish  (Fundulus stellifer) | Interspecific | nu and mt DNA seq | Mississippi River; Other Riverine | None | Hundt et al., 2017 |
| Blackstripe Topminnow  (Fundulus notatus)  Broadstripe Topminnow  (Fundulus euryzonus)  Blackspotted Topminnow  (Fundulus olivaceus) | Both | GBS | Other Riverine | Dated between populations for info on migration | Duvernell et al., 2019 |
| Redeye Bass  (Micropterus coosae) | Intraspecific | mt DNA seq | Other riverine | None | Baker et al., 2013 |
| Gulf killifish  (Fundulus grandis) | Intraspecific | microsatellites | Other | None | Williams et al., 2008 |
| Naked Goby  (Gobiosoma bosc) | Intraspecific | mt and nu DNA seq | Atlantic-Gulf; Apalachicola River | A-G divergence: Miocene 8.07 [3.74-13.12] mya; Apa. River divergence: Pliocene 4.58 [2.06-7.51] mya | Mila et al., 2017 |
| Least Killifish  (Heterandria formosa) | Intraspecific | mt and nu DNA seq; allozymes | None- spp expansion and contraction | Species split 8.478 [3.939-14.913] mya Miocene; lineage diversification 61.6-20.4 [8.4-33.2] kya Pleistocene | Bagley et al., 2013 |
| Bigeye Chub  (Hybopsis amblops)  Pallid Shiner  (Hybopsis amnis)  Highback Chub  (Hybopsis hypsinotus)  Lined Chub  (Hybopsis lineapunctata)  Rosyface Chub  (Hybopsis rubrifrons)  Clear Chub  (Hybopsis winchelli) | Interspecific | mt DNA seq | Mississippi River | None | Berendzen et al., 2008 |
| Silver Carp  (Hypophthalmichthys molitrix)  Bighead Carp  (Hypophthalmichthys nobilis) | Both | microsatellites; mt DNA seq | None | None | Farrington et al., 2017 |
| Silver Carp  (Hypophthalmichthys molitrix)  Bighead Carp  (Hypophthalmichthys nobilis) | Both | microsatellites; mt DNA genotyping | None - need finer molecular markers and resolution | None | Farrington et al., 2017 |
| Channel Catfish  (Ictalurus punctatus) | Intraspecific | mt DNA seq | Atlantic-Gulf; Mississippi River | None | Padhi, 2013 |
| Inland Silverside  (Menidia beryllina) | Intraspecific | mt and nu DNA seq | Atlantic-Gulf (not monophyletic) (Bootstrap <70) | None | Oswald et al., 2009 |
| Redspot Chub  (Nocomis asper)  Hornyhead Chub  (Nocomis biguttatus)  Redtail Chub  (Nocomis effusus)  River Chub  (Nocomis micropogon)  Bull Chub  (Nocomis raneyi)  Bluehead Chub  (Nocomis leptocephalus) | Interspecific | mt and nu DNA seq | Mississippi River | 0.4 mya Pleistocene (from a previous paper) | Echelle et al., 2014 |
| Carmine Shiner  (Notropis percobromus)  Rosyface Shiner  (Notropis rubellus)  Highland Shiner  (Notropis micropteryx)  Rocky Shiner  (Notropis suttkusi) | Interspecific | mt DNA seq; previously published allozyme data | None - more complex; more sampling; greater mol. Resolution | within species complex 3.42-0.9 mya Pliocene-Pleistocene | Berendzen et al., 2008 |
| Slender Madtom  (Noturus exilis) | Intraspecific | mt and nu DNA seq; sanger | Mississippi River (pp = 1; Bootstrap = 100)r; Other riverine | late Miocene at 9.7 [5.32-14.93] mya; within the species spanned the late Miocene to mid-Pleistocene. | Blanton et al., 2013 |
| Stonecat  (Noturus flavus) | Intraspecific | mt DNA seq | Other; Laurentide Ice Sheet | None | Faber et al., 2009 |
| Blackbanded Darter  (Percina nigrofasciata)  Westfall’s Darter  (Percina westfalli)  Halloween Darter  (Percina crypta) | Interspecific | mt and nu DNA seq | Apalachicola and associated Rivers PP>90 | None | Hayes and Piller, 2018 |
| Shiners  (10 Pteronotropis species) | Interspecific | mt DNA seq | Atlantic-Gulf; Apalachicola River; Other Riverine | None | Mayden and Allen, 2015 |
| Flathead Catfish  (Pylodictis olivaris) | Intraspecific | mt DNA seq | Mississippi River (pp=1.0) | 11.27 [0.720-2.050] mya (Pleistocene); within lineages E=0.81-0.25; W= 0.56-0.12 | Padhi, 2014 |
| Creek Chub  (Semotilus atromaculatus) | Intraspecific | nu and mt DNA seq | Other Riverine | None | Schonhuth et al., 2018 |
| Dusky Pipefish  (Syngnathus ﬂoridae) | Intraspecific | mt DNA seq; microsatellites | Atlantic-Gulf (Bootstrap = 99,100) | None | Mobley et al., 2010 |
| Coyote  (Canis latrans) | Intraspecific | mt DNA seq | None - high dispersal distance; high adaptability; lack of structure | None | Koblmueller et al., 2012 |
| Southeastern Pocket Gopher  (Geomys pinetis) | Intraspecific | mt DNA seq | Apalachicola River (pp =?, Bootstrap = ?) | e-w lineage split 1.37 [1.9-0.830] mya Pleistocene | Soto-Centeno et al., 2013 |
| Eastern Red Bat  (Lasiurus borealis) | Intraspecific | microsatellites; mt DNA seq | None - indicates panmixia; high mobility but low use of migration paths; anthropogenic disturbance (wind turbines) | None | Vonhof and Russell, 2015 |
| Striped Skunk  (Mephitis mephitis) | Intraspecific | microsatellites and mt DNA seq | Mississippi River (pp=0.9348) | Pleistocene 130 [60-213] kya | Barton and Wisely, 2012 |
| Cotton Deermouse  (Peromyscus gossypinus)  White-footed Deermouse  (Peromyscus leucopus) | Interspecific | ddRADseq | Mississippi River | None | Delaney and Hoekstra, 2018 |
| Raccoon  (Procyon lotor) | Intraspecific | mt DNA seq | Mississippi River; Florida Peninsula | None | Cullingham et al., 2008 |
| Marsh Rabbit  (2 Sylvilagus palustris ssp) | Intraspecific | mt DNA seq and microsatellites | Florida Peninsula (pp=1. Bootstrap=98, not monophyletic) | None | Tursi et al., 2013 |
| Eastern Fox Squirrel  (Sciurus niger) | Intraspecific | mt DNA seq | None - high mobility and rapid range expansion | None | Moncrief et al., 2010 |
| Eastern Fox Squirrel  (Sciurus niger) | Intraspecific | mt DNA seq (Sc) | Laurentide Ice Sheet | None | Moncrief et al., 2012 |
| Eastern Gray Squirrel  (Sciurus carolinensis) |  |  |  |  |  |
| Eastern Spotted Skunk  (Spilogale putorius) | Intraspecific | microsatellites; mt DNA seq | E-W Miss River/ App Mts Bootstrap=98, PP=1; Peninsular FL Bootstrap <75, PP<0.95 | None | Shaffer et al., 2018 |
| American Black Bear  (Ursus americanus) | Intraspecific | Mt DNA; SNPs (RAD seq) | Mississippi River (pp=1); Laurentide Ice Sheet | 0.17 [0.12-0.23] mya Pleistocene | Puckett et al., 2015 |
| **VERTEBRATE (REPTILE)** |  |  |  |  |  |
| Northern Cottonmouth  (Agkistrodon piscivorus) | Intraspecific | AFLPs; mt DNA seq | None- greater resolution; need nu DNA seq; expand sampling | None | Strickland et al., 2014 |
| Eastern Copperhead  (Agkistrodon contortrix)  Northern Cottonmouth  (Agkistrodon piscivorus) | Intraspecific | mt DNA seq | Florida Peninsula (Bootstrap = 80), Other (A. piscivorus); Mississippi River (Bootstrap = 78), Other (A ontortrix) | Florida Peninsula 1.88 [1.46-2.31] mya, Other (A. piscivorus); Mississippi River 1.4 [1.01-1.79] mya, Other (A ontortrix) | Douglas et al., 2009 |
| Eastern Copperhead  (Agkistrodon contortrix)  Northern Cottonmouth  (Agkistrodon piscivorus) | Interspecific | mt DNA seq | None (A. contortrix) | Ac ~6.6 mya (Late Miocene); Ap ~5.3 mya (Late Miocene, Early Pliocene); Ap FL ~2.5 mya (Late Pliocene)  Ac 6.6 [4.84-8.53] mya (Late Miocene); Ap 5.3 [3.64-7.14] mya (Late Miocene, Early Pliocene); Ap FL 2.5 [1.17-3.97] mya (Late Pliocene) | Guiher and Burbrink, 2008 |
|  |  |  | Florida (pp= 1, Bootstrap = 96) (A. piscivorus) |  |  |
|  | Both | mt and nu DNA seq | Florida Peninsula | None | Burbrink and Guiher, 2015 |
| Green anole  (Anolis carolinensis) | Intraspecific | RADseq | Florida Peninsula (Bootstrap = 1); Atlantic-Gulf (1); Other: Carolinas (1), NW (1), E(), and S Florida(1) | Miocene-Pleistocene; species 12.87 [11.8-13.93] mya; FL Pen 6.64-2.78 [2.3-7.31] mya; G-A and Carolinas 2.01 [1.51-2.66] mya | Manthey et al., 2016 |
|  | Intraspecific | mt DNA seq | Florida Peninsula (pp=1. Bootstrap=99 not monophyletic) | ~6.8–12.6 MYA (mid-Miocene to the early Pliocene.) | Campbell-Staton et al., 2012 |
|  | Intraspecific | mt and nu DNA seq; Sanger | Other | None | Tollis et al., 2012 |
|  | Intraspecific | mt and nu DNA seq | Atlantic-Gulf | Species split 9.3 mya (Miocene); Within lineage 2.15-0.75 [0.45-3.1] mya (Pleistocene) | Tollis and Boissinot, 2014 |
|  | Intraspecific | WGS (TRU-seq) | Peninsular FL, Carolinas, Atlantic-Gulf in FL | None | Bourgeois et al., 2019 |
| Scarletsnake  (Cemophora coccinea)  Texas Scarletsnake  (Cemophora coccinea ssp. lineri) | Intraspecific | mt and nu DNA seq | Florida Peninsula | Species split: Pliocene 2.91 [4.86-1.37] mya | Weinell and Austin, 2017 |
| North American Racer  (Coluber constrictor) | Intraspecific | mt DNA seq | Mississippi River (pp= 1, Bootstrap = 100); Atlantic-Gulf (pp=1, Bootstrap = 95; Florida Peninsula (pp= 1, Bootstrap = 100) | E-W Miss. Rvr 6.09 mya; FL peninsula 6.183 [3.163-9.667] mya; Atlantic-Gulf 3.45 [1.715-5.371] mya | Burbrink et al., 2008 |
| Ring-necked Snake  (Diadophis punctatus) | Intraspecific | mt DNA seq | Mississippi River; (pp = 1, Bootstrap = 86) Florida Peninsula (pp = 1, Bootstrap = 100) | E-W Miss Rvr 2.461 [0.998-4.172] mya; FL peninsula 2.721 [0.953-4.88] mya | Fontanella et al., 2008 |
|  | Intraspecific | mt DNA seq | None- high diversity; unclear (sub)species boundaries | Species spelit late Miocene 6.5 [3.15-10.46] mya | Fontanella and Siddall, 2010 |
| Eastern Indigo Snake  (Drymarchon couperi)  Western Indigo Snake  (Drymarchon melanurus) | Interspecific | mt and nu DNA seq | Atlantic-Gulf (D. couperi) | Species divergence 5.9 mys Mio-Plio; G-A split 2.0 mya Plio-Pleis | Krysko et al., 2016 |
| Eastern Indigo Snake  (Drymarchon couperi) | Intraspecific | microsatellite, mt and nu DNA seq | Atlantic-Gulf (Bootstrap=80) | None | Folt et al., 2019 |
| Speckled Kingsnake  (Lampropeltis holbrooki)  Eastern Black Kingsnake  (Lampropeltis nigra)  Outer Banks kingsnake  (Lampropeltis getula “sticticeps”)  Eastern Kingsnake  (Lampropeltis getula getula)  Florida Kingsnake  (Lampropeltis getula floridana)  Apalachicola Kingsnake  (Lampropeltis getula meansi) | Interspecific | mt and nu DNA seq | Apalachicola River (pp=.99); Florida Peninsula (pp=0.99) ; Appalachian Mountains (?) | Pliocene - Pleistocene; 0.32 [0.5-0.1] mya Apalachicola River split; 0.4 [0.7-0.1] Mississippi River Split; 0.54 [0.8-0.2] mya FL Pen. Split; 2.0 for App Mts split; 4.7 [6.8-2.7] mya for whole complex | Krysko et al., 2017 |
| Eastern Kingsnake  (Lampropeltis getula)  Yellow-bellied Kingsnake  (Lampropeltis calligaster)  Mole Kingsnake  (Lampropeltis rhombomaculata)  South Florida Mole Kingsnake  (Lampropeltis occipitolineata) | Intraspecific | DNA seq | Mississippi River; Appalachian Mountains; Chattahoochee and Apalachicola Rivers | early Pliocene 4.91 [2.63-7.32] mya | Pyron and Burbrink, 2009 |
|  | Interspecific | mt and nu DNA seq | Mississippi River (pp=1); Florida Peninsula(pp=0.8) | Pleistocene 1.56 [0.64-2.92] mya Mississippi River, 0.88 [0.003-1.72] mya FL Pen Pen) | McKelvy and Burbrink, 2017 |
| Diamondback Terrapins  (Malaclemys terrapin) | Intraspecific | microsatellites | Atlantic-Gulf; Other | None | Converse et al., 2017 |
| Diamond-backed Watersnake  (Nerodia rhombifer) | Intraspecific | mt DNA seq | Mississippi River (pp=0.99) | 1.39 [0.84-1.99] mya (Pleistocene) | Brandley et al., 2010 |
| Plain-bellied Watersnake  (Nerodia erythrogaster) | Intraspecific | mt DNA seq | None- greater sampling; need to use nu DNA markers | None | Makowsky et al., 2010 |
| Cooters  (9 Pseudemys species) | Interspecific | mt DNA seq | None- retained polymorphisms; recent hybridization; add in nu DNA; date divergence | None | Jackson et al., 2012 |
| Eastern Fence Lizard  (Sceloporus undulatus)  Prairie Lizard  (Sceloporus consobrinus)  Florida Scrub Lizard  (Sceloporus woodi) | Interspecific | nu DNA seq | Mississippi River; Florida peninsula | None | Leache, 2009 |
| Ground Skink  (Scincella lateralis) | Intraspecific | nu DNA seq | Mississippi River (pp=.95); Apalachicola River (pp=.98) | None | Jackson and Austin, 2012 |
| Dekay's Brownsnake  (Storeria dekayi)  Red-bellied Snake  (Storeria occipitomaculata)  Florida Brownsnake  (Storeria victa) | Both | SNPs (Illumina Hi-seq) | Florida Peninsula; Mississippi River | None | Pyron et al., 2016 |
| Gulf Coast Box Turtle  (Terrapene carolina major)  Florida Box Turtle  (Terrapene carolina bauri)  Eastern Box Turtle  (Terrapene carolina carolina)  Three-toed Box Turtle  (Terrapene carolina triunguis) | Intraspecific | mt DNA seq; microsatellites | Mississippi River; Florida peninsula | None | Butler et al., 2011 |
|  | Intraspecific | mt and nu DNA seq | Mississippi River (pp=0.98); Apalachicola River (pp=1.0, Bootstrap = 99); Other Riverine | None | Jackson and Austin, 2010 |
| Box Turtles  (7 Terrapene species) | Interspecific | mt and nu DNA seq | None- more sampling; finer resolution; species boundaries question | mid-late Miocene 15.3 [19.5-11.8] mya | Martin et al., 2013 |
| Loggerhead Musk Turtle  (Sternotherus minor minor)  Stripe-Necked Musk Turtle  (Sternotherus minor peltifer)  Eastern Musk Turtle  (Sternotherus odoratus)  Razor-backed Musk Turtle  (Sternotherus carinatus)  Flattened Musk Turtle  (Sternotherus depressus) | Both | 3RAD seq; mt DNA sequence | Other Riverine | None | Scott et al., 2018 |

*Table S2:* Summary of discontinuities observed by taxon in the present study. Number in parentheses is the percentage of studies out of total studies for each taxon. Note that many studies, particularly those that focused on several species, showed several patterns, such that the total number adds to more than the total number of papers examined.

|  | **Invertebrate** | | | | **Vertebrate** | | | | | | **Plant** | | | **Other** | **Total** |
| --- | --- | --- | --- | --- | --- | --- | --- | --- | --- | --- | --- | --- | --- | --- | --- |
| **Discontinuity** | Arthropod | Crustacean | | Mollusk | Amphibian | | Bird | Fish | Mammal | Reptile | Angiosperm | Gymnosperm | Liverwort | Fungi |  |
| Atlantic - Gulf Coast | 4  (11%) | 2  (100%) | 1  (14%) | | 2  (8%) | 1  (8%) | | 8  (19%) | 0 | 7  (13%) | 6  (8%) | 0 | 0 | 0 | 31 |
|  | 4  (11%) | 0 | 1  (14%) | | 3  (12%) | 1  (8%) | | 3  (7%) | 1  (6%) | 4  (8%) | 2  (3%) | 0 | 0 | 0 | 19 |
| Mississippi River | 4  (11%) | 0 | 1  (14%) | | 3  (12%) | 0 | | 10  (24%) | 5  (31%) | 11  (21%) | 12  (17%) | 0 | 0 | 0 | 46 |
| Appalachian Mountains | 6  (17%) | 0 | 0 | | 2  (8%) | 2  (17%) | | 0 | 1  (6%) | 2  (4%) | 10  (14%) | 0 | 0 | 0 | 23 |
| Appalachian Mountains/ Apalachicola River & Mississippi River | 1  (3%) | 0 | 0 | | 0 | 0 | | 0 | 1  (6%) | 0 | 1  (1%) | 0 | 0 | 0 | 3 |
| Peninsular Florida | 2  (6%) | 0 | 0 | | 3  (12%) | 4  (33%) | | 2  (5%) | 3  (19%) | 14  (27%) | 6  (8%) | 2  (50%) | 0 | 0 | 36 |
| Laurentide Ice Sheet | 4  (11%) | 0 | 0 | | 0 | 0 | | 1  (2%) | 2  (13%) | 0 | 10  (14%) | 0 | 0 | 0 | 17 |
| Other riverine | 4  (11%) | 0 | 1  (14%) | | 4  (16%) | 0 | | 10  (24%) | 0 | 2  (4%) | 2  (3%) | 0 | 0 | 0 | 23 |
| Other | 4  (11%) | 0 | 1  (14%) | | 3  (12%) | 0 | | 3  (7%) | 0 | 6  (12%) | 4  (6%) | 2  (50%) | 0 | 0 | 23 |
| None | 3  (8%) | 0 | 2  (29%) | | 5  (20%) | 4  (33%) | | 5  (12%) | 3  (19%) | 6  (12%) | 18  (25%) | 0 | 1  (100%) | 2  (100%) | 49 |

## Appendix S1 Literature Cited in Table S1

Acosta, J. J., Fahrenkrog, A. M., Neves, L. G., Resende, M. F. R., Dervinis, C., Davis, J. M.,

Holliday, J. A., Kirst, M.. 2019. Exome Resequencing Reveals Evolutionary History, Genomic Diversity, and Targets of Selection in the Conifers *Pinus taeda* and *Pinus elliottii*. Genome and Biology and Evolution 11:508-520.

Austin, James D., Zamudio, K. R.. 2008. Incongruence in the Pattern and Timing of Intra-

Specific Diversification in Bronze Frogs and Bullfrogs (Ranidae). Molecular Phylogenetics and Evolution 48:1041-1053.

Baeza, J. A., Fuentes, M. S.. 2013. Phylogeography of the Shrimp *Palaemon floridanus*

(Crustacea: Caridea: Palaemonidae): A Partial Test of Meta-Population Genetic Structure in the Wider Caribbean. Marine Ecology 34:381-393.

Baeza, J. A., Prakash, S.. 2019. an Integrative Taxonomic and Phylogenetic Approach

Reveals a Complex of Cryptic Species in the "Peppermint' Shrimp *Lysmata wurdemanni* Sensu Stricto. Zoological Journal of the Linnean Society 185:1018-1038.

Bagley, J. C., Sandel, M., Travis, J., De Lourdes Lozano-Vilano, M., Johnson, J. B.. 2013.

Paleoclimatic Modeling and Phylogeography of Least Killifish, *Heterandria formosa*: Insights into Pleistocene Expansion-Contraction Dynamics and Evolutionary History of North American Coastal Plain Freshwater Biota. BMC Evolutionary Biology 13:223.

Bagley, R. K., Sousa, V. C., Niemiller, M. L., Linnen, C. R.. 2017. History, Geography and Host

Use Shape Genomewide Patterns of Genetic Variation in the Redheaded Pine Sawfly (*Neodiprion lecontei*). Molecular Ecology 26:1022-1044.

Baker, W. H., Blanton, R. E., Johnston, C. E.. 2013. Diversity Within the Redeye Bass,

*Micropterus coosae* (Perciformes: Centrarchidae) Species Group, with Descriptions of Four New Species. Zootaxa 3635:379-401.

Barnard-Kubow, K. B., Debban, C. L., Galloway, L. F.. 2015. Multiple Glacial Refugia Lead to

Genetic Structuring and the Potential for Reproductive Isolation in a Herbaceous Plant. American Journal of Botany 102:1842-1853.

Barrow, L. N., Soto-Centeno, J. A., Warwick, A. R., Lemmon, A. R., and Lemmon, E. M.. 2017.

Evaluating Hypotheses of Expansion from Refugia Through Comparative Phylogeography of South-Eastern Coastal Plain Amphibians. Journal of Biogeography 44:2692-2705

Barrowclough, G. F., Groth, J. G., Bramlett, E. K., Lai, J. E., Mauck, W. M.. 2018.

Phylogeography and Geographic Variation in the Red-Bellied Woodpecker (*Melanerpes carolinus*): Characterization of mtDNA and Plumage Hybrid Zones. Wilson Journal of Ornithology 130: 671-683

Barrowclough, G. F., Groth, J. G., Mauck, W. M., Blair, M. E.. 2019. Phylogeography and

Species Limits in the Red-Shouldered Hawk (*Buteo lineatus*): Characterization of the Northern Florida Suture Zone in Birds. Ecology and Evolution 9:6245-6258

Barrowclough, G. F., Groth, J. G., Odom, K. J., Lai, J. E.. 2011. Phylogeography of the Barred

Owl (*Strix varia*): Species Limits, Multiple Refugia, and Range Expansion. Auk 128:696-706.

Barton, H. D., Wisely, S. M.. 2012. Phylogeography of Striped Skunks (*Mephitis mephitis*) in

North America: Pleistocene Dispersal and Contemporary Population Structure. Journal of Mammalogy 93:38-51.

Beamer, D. A., Lamb, T. 2008. Dusky Salamanders (Desmognathus, Plethodontidae) from the

Coastal Plain: Multiple Independent Lineages and Their Bearing on the Molecular Phylogeny of the Genus. Molecular Phylogenetics and Evolution 47:143-153.

Bemmels, J., Dick, C.. 2018. Genomic Evidence of a Widespread Southern Distribution During

the Last Glacial Maximum for Two Eastern North American Hickory Species. Journal of Biogeography 45:1739-1750

Bentley, K. E., Mauricio, R.. 2016. High Degree of Clonal Reproduction and Lack of Large-

Scale Geographic Patterning Mark the Introduced Range of the Invasive Vine, Kudzu (Pueraria Montana Var. Lobata), in North America. American Journal of Botany 103:1499-1507.

Berendzen, P. B., Gamble, T., Simons, A. M.. 2008. Phylogeography of the Bigeye Chub

*Hybopsis amblops* (Teleostei: Cypriniformes): Early Pleistocene Diversification and Post-Glacial Range Expansion. Journal of Fish Biology 73:2021-2039.

Berendzen, P. B., Simons, A. M., Wood, R. M., Dowling, T. E., Secor, C. L.. 2008. Recovering

Cryptic Diversity and ancient Drainage Patterns in Eastern North America: Historical Biogeography of the *Notropis rubellus* Species Group (Teleostei : Cypriniformes). Molecular Phylogenetics and Evolution 46:721-737.

Blanton, R. E., Page, L. M., Hilber, S. A.. 2013. Timing of Clade Divergence and Discordant

Estimates of Genetic and Morphological Diversity in the Slender Madtom, Noturus Exilis (Ictaluridae). Molecular Phylogenetics and Evolution 66:679-693.

Blum, M. J., Bando, K. J., Katz, M., Strong, D. R.. 2007. Geographic Structure, Genetic

Diversity and Source Tracking of *Spartina alterniflora*. Journal of Biogeography 34: 2055-2069.

Blum, M. J., Neely, D. A., Harris, P. M., Mayden, R. L.. 2008. Molecular Systematics of the

Cyprinid Genus *Campostoma* (Actinopterygii : Cypriniformes): Disassociation Between Morphological and Mitochondrial Differentiation. Copeia 2: 360-369.

Bossu, C. M., Beaulieu, J. M., Ceas, P. A., Near, T. J.. 2013. Explicit Tests of Palaeodrainage

Connections of Southeastern North America and the Historical Biogeography of Orangethroat Darters (Percidae: *Etheostoma*: *Ceasia*). Molecular Ecology 22:5397-5417.

Bossu, C. M., Near, T. J.. 2009. Gene Trees Reveal Repeated Instances of Mitochondrial DNA

Introgression in Orangethroat Darters (Percidae: *Etheostoma*). Systematic Biology 58:114-129.

Bourgeois, Y., Ruggiero, R. P., Manthey, J. D., Boissinot, S.. 2019. Recent Secondary Contacts,

Linked Selection, and Variable Recombination Rates Shape Genomic Diversity in the Model Species *Anolis carolinensis*. Genome Biology and Evolution 11:2009-2022

Brandley, M. C., Guiher, T. J., Pyron, R. A., Winne, C. T., Burbrink, F. T.. 2010. Does Dispersal

Across an Aquatic Geographic Barrier Obscure Phylogeographic Structure in the Diamond-Backed Watersnake (*Nerodia rhombifer*)?. Molecular Phylogenetics and Evolution 57:552-560.

Burbrink, F. T., Fontanella, F., Pyron, R. A., Guiher, T. J., Jimenez, C.. 2008. Phylogeography

Across a Continent: the Evolutionary and Demographic History of the North American Racer (Serpentes : Colubridae : *Coluber constrictor*). Molecular Phylogenetics and Evolution 47:274-288.

Burbrink, F. T., Guiher, T. J.. 2015. Considering Gene Flow When Using Coalescent Methods to

Delimit Lineages of North American Pitvipers of the Genus *Agkistrodon*. Zoological Journal of the Linnean Society 173:505-526.

Butler, J. M., Dodd, C. K., Jr., Aresco, M., Austin, J. D.. 2011. Morphological and Molecular

Evidence Indicates That the Gulf Coast Box Turtle (*Terrapene carolina major*) Is Not a Distinct Evolutionary Lineage in the Florida Panhandle. Biological Journal of the Linnean Society 102:889-901.

Campbell-Staton, S. C., Goodman, R. M., Backstroem, N., Edwards, S. V., Losos, J. B., Kolbe,

J. J.. 2012. Out of Florida: mtDNA Reveals Patterns of Migration and Pleistocene Range Expansion of the Green anole Lizard (*Anolis carolinensis*). Ecology and Evolution 2:2274-2284.

Campitelli, B. E., Stinchcombe, J. R.. 2014. Population Dynamics and Evolutionary History of

the Weedy Vine *Ipomoea hederacea* in North America. G3-Genes Genomes Genetics 4:1407-1416.

Carstens, B. C., Satler, J. D.. 2013. the Carnivorous Plant Described as *Sarracenia alata*

Contains Two Cryptic Species. Biological Journal of the Linnean Society 107:737-746.

Cavender-Bares, J., Gonzalez-Rodriguez, A., Eaton, D. A. R., Hipp, A. A. L., Beulke, A.,

Manos, P. S.. 2015. Phylogeny and Biogeography of the American Live Oaks (*Quercus* Subsection *Virentes*): A Genomic and Population Genetics Approach. Molecular Ecology 24:3668-3687.

Cerame, B., Cox, J. A., Brumfield, R. T., Tucker, J. W., Taylor, S. S.. 2014. Adaptation to

Ephemeral Habitat May Overcome Natural Barriers and Severe Habitat Fragmentation in a Fire-Dependent Species, the Bachman's Sparrow (*Peucaea aestivalis*). PLoS ONE 9:105782.

Chabarria, R. E., Murray, C. M., Moler, P. E., Bart, H. L., Jr., Crother, B. I., Guyer, C.. 2018.

Evolutionary Insights into the North American *Necturus beyeri* Complex (Amphibia: Caudata) Based on Molecular Genetic and Morphological analyses. Journal of Zoological Systematics and Evolutionary Research 56:352-363

Chong, J. P., Harris, J L., Roe, K J.. 2016. Incongruence Between mtDNA and Nuclear Data in

the Freshwater Mussel Genus *Cyprogenia* (Bivalvia: Unionidae) and its Impact on Species Delineation. Ecology and Evolution 6:2439-2452.

Converse, P. E., Kuchta, S. R., Hauswaldt, J. S., Roosenburg, W. M.. 2017. Turtle Soup,

Prohibition, and the Population Genetic Structure of Diamondback Terrapins (*Malaclemys terrapin*). PLoS ONE 12:0181898.

Cullingham, C. I., Kyle, C. J., Pond, B. A., White, B. N.. 2008. Genetic Structure of Raccoons in

Eastern North America Based on mtDNA: Implications for Subspecies Designation and Rabies Disease Dynamics. Canadian Journal of Zoology 86:947-958.

Deane, P. E., Mccoy, K. D., Robertson, R. J., Birt, T. P., Friesen, V. L.. 2013. Minimal Genetic

Structure in the Cerulean Warbler Despite Evidence for Ecological Differentiation Among Populations. Condor 115:178-185.

Degner, J. F., Silva, D. M., Hether, T. D., Daza, J. M., Hoffman, E. A.. 2010. Fat Frogs, Mobile

Genes: Unexpected Phylogeographic Patterns for the Ornate Chorus Frog (*Pseudacris ornata*). Molecular Ecology 19:2501-2515.

Delaney, E. K., Hoekstra, H. E.. 2018. Sexual Imprinting and Speciation Between Two

*Peromyscus* Species. Evolution 72:274-287

Desaix, M. G., Bulluck, L. P., Eckert, A. J., Viverette, C. B., Boves, T. J., Reese, J. A., Tonra, C.

M., Dyer, Rodney J.. 2019. Population Assignment Reveals Low Migratory Connectivity in a Weakly Structured Songbird. Molecular Ecology 28:2122-2135

Douglas, M. E., Douglas, M. R., Schuett, G. W., Porras, L. W.. 2009. Climate Change and

Evolution of the New World Pitviper Genus *Agkistrodon* (Viperidae). Journal of Biogeography 36:1164-1180.

Douglas, N. A., Wall, W. A., Xiang, Qiu-Yun (Jenny), Hoffmann, W. A., Wentworth, T. R.,

Gray, J. B., Hohmann, M. G.. 2011. Recent Vicariance and the Origin of the Rare, Edaphically Specialized Sandhills Lily, *Lilium pyrophilum* (Liliaceae): Evidence from Phylogenetic and Coalescent analyses. Molecular Ecology 20:2901-2915.

Duvernell, D. D., Westhafer, E., Schaefer, J. F.. 2019. Late Pleistocene Range Expansion of

North American Topminnows Accompanied by Admixture and Introgression. Journal of Biogeography 46:2126-2140

Echelle, A. A., Schwemm, M. R., Lang, N. J., Nagle, B. C., Simons, A. M., Unmack, P. J.,

Fisher, W. L., Hoagstrom, C. W.. 2014. Molecular Systematics and Historical Biogeography of the *Nocomis biguttatus* Species Group (Teleostei: Cyprinidae): Nuclear and Mitochondrial Introgression and a Cryptic Ozark Species. Molecular Phylogenetics and Evolution 81:109-119.

Echt, C. S., Demeer, D., Gustafson, D.. 2011. Patterns of Differentiation Among Endangered

Pondberry Populations. Conservation Genetics 12:1015-1026.

Eckert, A. J., Van Heerwaarden, J., Wegrzyn, J. L., Nelson, C. Dana, Ross-Ibarra, Jeffrey,

Gonzalez-Martinez, Santiago C., Neale, David. B.. 2010. Patterns of Population Structure and Environmental Associations to Aridity Across the Range of Loblolly Pine (*Pinus taeda* L., Pinaceae). Genetics 185:969-982.

Eo, S. H., Wares, J. P., Carroll, J. P.. 2010. Subspecies and Units for Conservation and

Management of the Northern Bobwhite in the Eastern United States. Conservation Genetics 11:867-875.

Escudero, M., Lovit, M., Brown, B. H., Hipp, A. L.. 2019. Rapid Plant Speciation Associated

with the Last Glacial Period: Reproductive Isolation and Genetic Drift in Sedges. Botanical Journal of the Linnean Society 190:303-314.

Faber, J. E., Rybka, J., White, M. M.. 2009. Intraspecific Phylogeography of the Stonecat

Madtom, *Noturus flavus.* Copeia 3:563-571.

Fahrenkrog, A. M., Neves, L. G., Resende, M. F. R., Dervinis, C., Davenport, R., Barbazuk, W.

B., Kirst, M.. 2017. Population Genomics of the Eastern Cottonwood (*Populus deltoides*). Ecology and Evolution 7:9426-9440

Farrington, H. L., Edwards, C. E., Bartron, M., Lance, R. F.. 2017. Phylogeography and

Population Genetics of Introduced Silver Carp (*Hypophthalmichthys molitrix*) and Bighead Carp (*H. nobilis*) in North America. Biological Invasions 19:2789-2811.

Fehrmann, S., Philbrick, C. T., Halliburton, R.. 2012. Intraspecific Variation in *Podostemum*

*ceratophyllum* (Podostemaceae): Evidence of Refugia and Colonization Since the Last Glacial Maximum. American Journal of Botany 99:145-151.

Fetter, K. C., Weakley, A.. 2019. Reduced Gene Flow from Mainland Populations of

*Liriodendron tulipifera* into the Florida Peninsula Promotes Diversification. International Journal of Plant Sciences 180:253-269

Fisher, J. R., Fisher, D. M., Skvarla, M. J., Nelson, W. A., Dowling, A. P. G.. 2017. Revision of

Torrent Mites (*Parasitengona, Torrenticolidae, Torrenticola*) of the United States and Canada: 90 Descriptions, Molecular Phylogenetics, and a Key to Species. Zookeys 1-496.

Folt, B., Bauder, J., Spear, S., Stevenson, D., Hoffman, M., Oaks, J. R., Wood, P. L., Jr., Jenkins,

C., Steen, D. A., Guyer, C.. 2019. Taxonomic and Conservation Implications of Population Genetic Admixture, Mito-Nuclear Discordance, and Male-Biased Dispersal of a Large Endangered Snake, *Drymarchon couperi*. PLoS ONE 14:0214439

Folt, B., Garrison, N., Guyer, C., Rodriguez, J., Bond, J. E.. 2016. Phylogeography and

Evolution of the Red Salamander (*Pseudotriton ruber*). Molecular Phylogenetics and Evolution 98:97-110.

Fontanella, F. M., Feldman, C. R., Siddall, M. E., Burbrink, F. T.. 2008. Phylogeography of

*Diadophis punctatus*: Extensive Lineage Diversity and Repeated Patterns of Historical Demography in a Trans-Continental Snake. Molecular Phylogenetics and Evolution 46:1049-1070.

Fontanella, F. M., Garner, Y., Starnes, J., Whitaker, M.. 2019. Evidence for Panmixia Despite

Barriers to Gene Flow in the Hooked Mussel, *Ischadium recurvum* (Mytilidae, Brachidontinae) Along the North American Coastline. Mitochondrial DNA Part A 30:75-81.

Fontanella, F., Siddall, M. E.. 2010. Evaluating Hypotheses on the Origin and Diversification of

the Ringneck Snake *Diadophis punctatus* (Colubridae: Dipsadinae). Zoological Journal of the Linnean Society 158:629-640.

Germain-Aubrey, C. C., Soltis, P. S., Neubig, K. M., Thurston, T., Soltis, D, E., Gitzendanner,

M. A.. 2014. Using Comparative Biogeography to Retrace the Origins of an Ecosystem: The Case of Four Plants Endemic to the Central Florida Scrub. International Journal of Plant Sciences 175:418-431.

Glotzbecker, G. J., Alda, F., Broughton, R. E., Neely, D. A., Mayden, R. L., Blum, M. J.. 2016.

Geographic Independence and Phylogenetic Diversity of Red Shiner Introductions. Conservation Genetics 17:795-809.

Gonzales, E., Hamrick, J. L., Chang, Shu-Mei. 2008. Identification of Glacial Refugia in South-

Eastern North America By Phylogeographical analyses of a Forest Understorey Plant, *Trillium cuneatum*. Journal of Biogeography 35:844-852.

Grabowski, P. P., Morris, G. P., Casler, M. D., Borevitz, J. O.. 2014. Population Genomic

Variation Reveals Roles of History, Adaptation and Ploidy in Switchgrass. Molecular Ecology 23:4059-4073.

Graham, Sean P., Kline, Richard, Steen, David A., Kelehear, Crystal. 2018. Description of an

Extant Salamander from the Gulf Coastal Plain of North America: The Reticulated Siren, *Siren reticulata*. PLoS ONE 13: 0207460.

Gray, D. A., Huang, H., Knowles, L. L.. 2008. Molecular Evidence of a Peripatric Origin for

Two Sympatric Species of Field Crickets (*Gryllus rubens* and *G. texensis*) Revealed from Coalescent Simulations and Population Genetic Tests. Molecular Ecology 17:3836-3855.

Guiher, T. J., Burbrink, F. T.. 2008. Demographic and Phylogeographic Histories of Two

Venomous North American Snakes of the Genus *Agkistrodon*. Molecular Phylogenetics and Evolution 48:543-553.

Hadziabdic, D., Fitzpatrick, B. M., Wang, X., Wadl, P. A., Rinehart, T. A., Ownley, B. H.,

Windham, M. T., Trigiano, R. N.. 2010. Analysis of Genetic Diversity in Flowering Dogwood Natural Stands Using Microsatellites: The Effects of Dogwood Anthracnose. Genetica 138:1047-1057.

Halas, D., Simons, A. M.. 2014. Cryptic Speciation Reversal in the *Etheostoma zonale*

(Teleostei: Percidae) Species Group, with an Examination of the Effect of Recombination and Introgression on Species Tree Inference. Molecular Phylogenetics and Evolution 70:13-28.

Hamlin, J. A. P., Arnold, M. L.. 2014. Determining Population Structure and Hybridization for

Two Iris Species. Ecology and Evolution 4:743-755.

Hamlin, J. A. P., Arnold, M. L.. 2015. Neutral and Selective Processes Drive Population

Differentiation for *Iris hexagona*. Journal of Heredity 106:628-636.

Hayes, K. A., Karl, S. A.. 2009. Phylogenetic Relationships of Crown Conchs (*Melongena* Spp.):

The *Corona* Complex Simplified. Journal of Biogeography 36:28-38.

Hayes, M. M., Piller, K. R.. 2018. Patterns of Diversification in a North American Endemic Fish,

the Blackbanded Darter (Perciformes, Percidae). Zoologica Scripta 47:477-485

Hedin, M., McCormack, M.. 2017. Biogeographical Evidence for Common Vicariance and Rare

Dispersal in a Southern Appalachian Harvestman (Sabaconidae, *Sabacon cavicolens*). Journal of Biogeography 44:1665-1678.

Herman, T. A., Bouzat, J. L.. 2016. Range-Wide Phylogeography of the Four-Toed Salamander:

Out of Appalachia and into the Glacial Aftermath. Journal of Biogeography 43:666-678.

Hill, J. G.. 2015. Revision and Biogeography of the Melanoplus Scudderi Species Group

(Orthoptera: Acrididae: Melanoplinae) with a Description of 21 New Species and Establishment of the *Carnegiei* and *Davisi* Species Groups. Transactions of the American Entomological Society 141:252-350.

Hodel, R. G. J., Chen, S., Payton, A. C., McDaniel, S. F., Soltis, P., Soltis, D. E.. 2017. Adding

Loci Improves Phylogeographic Resolution in Red Mangroves Despite Increased Missing Data: Comparing Microsatellites and Rad-Seq and Investigating Loci Filtering. Scientific Reports 7:17598.

Hodel, R. G. J., De Souza Cortez, M. B., Soltis, P. S., Soltis, D. E.. 2016. Comparative

Phylogeography of Black Mangroves (*Avicennia germinan*s) and Red Mangroves (*Rhizophora mangle*) in Florida: Testing the Maritime Discontinuity in Coastal Plants. American Journal of Botany 103:730-739.

Hodel, R. G., Gonzales, E.. 2013. Phylogeography of Sea Oats (*Uniola paniculata*), a Dune-

Building Coastal Grass in Southeastern North America. Journal of Heredity 104:656-665.

Hundt, P. J., Berendzen, P. B., Simons, A. M.. 2017. Species Delimitation and Phylogeography

of the Studfish *Fundulus catenatus* Species Group (Ovalentaria: Cyprinodontiformes). Zoological Journal of the Linnean Society 180:461-474.

Hyseni, C., Garrick, R. C.. 2019. The Role of Glacial-Interglacial Climate Change in Shaping the

Genetic Structure of Eastern Subterranean Termites in the Southern Appalachian Mountains, USA. Ecology and Evolution 9:4621-4636

Ikezaki, Y., Suyama, Y., Middleton, B. A., Tsumura, Y., Teshima, K., Tachida, H., Kusumi, J..

2016. Inferences of Population Structure and Demographic History for *Taxodium distichum*, a Coniferous Tree in North America, Based on Amplicon Sequencing Analysis. American Journal of Botany 103:1937-1949.

Inoue, K., Monroe, E. M., Elderkin, C. L., Berg, D. J.. 2014. Phylogeographic and Population

Genetic Analyses Reveal Pleistocene Isolation Followed by High Gene Flow in a Wide Ranging, But Endangered, Freshwater Mussel. Heredity 112:282-290.

Jackson, Nathan D., Austin, Christopher C.. 2010. The Combined Effects of Rivers and Refugia

Generate Extreme Cryptic Fragmentation Within the Common Ground Skink (*Scincella lateralis*). Evolution 64:409-428.

Jackson, N. D., Austin, C. C.. 2012. Inferring the Evolutionary History of Divergence Despite

Gene Flow in a Lizard Species, *Scincella lateralis* (Scincidae), Composed of Cryptic Lineages. Biological Journal of the Linnean Society 107:192-209.

Jackson, T. G., Jr., Nelson, D. H., Morris, A. B.. 2012. Phylogenetic Relationships in the North

American Genus *Pseudemys* (Emydidae) Inferred from Two Mitochondrial Genes. Southeastern Naturalist 11:297-310.

Kaufman, E. L., Stone, N. E., Scoles, G. A., Hepp, C. M., Busch, J. D., Wagner, D. M.. 2018.

Range-Wide Genetic Analysis of *Dermacentor variabilis* and its *Francisella*-Like Endosymbionts Demonstrates Phylogeographic Concordance Between Both Taxa. Parasites & Vectors 11:306.

Kennedy, J. P., Garavelli, L., Truelove, N. K., Devlin, D. J., Box, S. J., Cherubin, L. M., Feller,

I. C.. 2017. Contrasting Genetic Effects of Red Mangrove (*Rhizophora mangle* L.) Range Expansion Along West and East Florida. Journal of Biogeography 44:335-347.

Kiefer, C., Dobes, C., Koch, M. A.. 2009. *Boechera* Or Not? Phylogeny and Phylogeography of

Eastern North American *Boechera* Species (Brassicaceae). Taxon 58:1109-1121.

Koblmueller, S., Wayne, R. K., Leonard, J. A.. 2012. Impact of Quaternary Climatic Changes

and Interspecific Competition on the Demographic History of a Highly Mobile Generalist Carnivore, the Coyote. Biology Letters 8:644-647.

Koopman, M. M., Carstens, B. C.. 2010. Conservation Genetic Inferences in the Carnivorous

Pitcher Plant *Sarracenia alata (*Sarraceniaceae). Conservation Genetics 11:2027-2038.

Krysko, K. L., Nunez, C. A., Lippi, C. A., Smith, D. J., Granatosky, M. C.. 2016. Pliocene-

Pleistocene Lineage Diversifications in the Eastern Indigo Snake (*Drymarchon couperi*) in the Southeastern United States. Molecular Phylogenetics and Evolution 98:111-122.

Krysko, K. L., Nunez, L. P., Newman, C. E., Bowen, B. W.. 2017. Phylogenetics of Kingsnakes,

*Lampropeltis getula* Complex (Serpentes: Colubridae), in Eastern North America. Journal of Heredity 108:226-238.

Kuchta, S. R., Haughey, M., Wynn, A. H., Jacobs, J. F., Highton, R.. 2016. Ancient River

Systems and Phylogeographical Structure in the Spring Salamander, *Gyrinophilus porphyriticus*. Journal of Biogeography 43:639-652.

Lait, L. A., Hebert, P. D. N.. 2018. Phylogeographic Structure in Three North American Tent

Caterpillar Species (Lepidoptera: Lasiocampidae): *Malacosoma americana*, *M. californica,* and *M. disstria*. PeerJ 6:4479.

Lang, N. J., Echelle, A. A.. 2011. Novel Phylogeographic Patterns in a Lowland Fish,

*Etheostoma Ppoeliare* (Percidae). Southeastern Naturalist 10:133-144.

Lavretsky, P., Hernandez-Banos, B. E., Peters, J. L.. 2014. Rapid Radiation and Hybridization

Contribute to Weak Differentiation and Hinder Phylogenetic Inferences in the New World Mallard Complex (*Anas* spp.). Auk 131:524-538.

Leache, A. D.. 2009. Species Tree Discordance Traces to Phylogeographic Clade Boundaries in

North American Fence Lizards (*Sceloporus*). Systematic Biology 58:547-559.

Lendemer, J. C.. 2016. A New Look at *Parmotrema madagascariaceum* and *P. xanthinum* in

North America. Journal of the Torrey Botanical Society 143:285-296.

Li, P., Li, M., Shi, Y., Zhao, Y., Wan, Y., Fu, C., Cameron, K. M.. 2013. Phylogeography of

North American Herbaceous *Smilax* (Smilacaceae): Combined AFLP and cpDNA Data Support a Northern Refugium in the Driftless Area. American Journal of Botany 100:801-814.

Li, X., Dane, F.. 2013. Comparative Chloroplast and Nuclear DNA analysis of Castanea Species

in the Southern Region of the USA. Tree Genetics & Genomes 9:107-116.

Lower, S. E., Stanger-Hall, K. F., Hall, D. W.. 2018. Molecular Variation Across Populations of

a Widespread North American Firefly, *Photinus pyralis*, Reveals That Coding Changes Do Not Underlie Flash Color Variation or Associated Visual Sensitivity. BMC Evolutionary Biology 18:129.

Majure, L. C., Judd, W.S., Soltis, P. S., Soltis, D. E.. 2012. Cytogeography of the *Humifusa*

Clade of *Opuntia* S.S. Mill. 1754 (Cactaceae, Opuntioideae, Opuntieae): Correlations with Pleistocene Refugia and Morphological Traits in a Polyploid Complex. Comparative Cytogenetics 6:53-77.

Makowsky, R., Chesser, J., Rissler, L. J.. 2009. A Striking Lack of Genetic Diversity Across the

Wide-Ranging Amphibian *Gastrophryne carolinensis* (Anura: Microhylidae). Genetica 135:169-183.

Makowsky, R., Marshall, J. C., Jr., McVay, J., Chippindale, P. T., Rissler, L. J.. 2010.

Phylogeographic analysis and Environmental Niche Modeling of the Plain-Bellied Watersnake (*Nerodia erythrogaster*) Reveals Low Levels of Genetic and Ecological Differentiation. Molecular Phylogenetics and Evolution 55:985-995.

Manthey, J. D., Tollis, M., Lemmon, A. R., Lemmon, E. M., Boissinot, S.. 2016. Diversification

in Wild Populations of the Model Organism *Anolis carolinensis*: A Genome-Wide Phylogeographic Investigation. Ecology and Evolution 6:8115-8125.

Marsico, T. D., Sauby, K. E., Brooks, C. P., Welch, M. E., Ervin, G. N.. 2015. Phylogeographic

Evidence for a Florida Panhandle-Peninsula Discontinuity in the Distribution of *Melitara prodenialis* Walker (Lepidoptera: Pyralidae), a Native Cactus-Boring Moth. Insect Conservation and Diversity 8:377-388.

Martin, B. T., Bernstein, N. P., Birkhead, R. D., Koukl, J. F., Mussmann, S. M., Placyk, J. S., Jr..

2013. Sequence-Based Molecular Phylogenetics and Phylogeography of the American Box Turtles (*Terrapene* spp.) with Support from DNA Barcoding. Molecular Phylogenetics and Evolution 68:119-134.

Martin, M. D., Olsen, M. T., Samaniego, J. A., Zimmer, E. A., Gilbert, M. Thomas, P.. 2016.

The Population Genomic Basis of Geographic Differentiation in North American Common Ragweed (*Ambrosia artemisiifolia* L.). Ecology and Evolution 6:3760-3771.

Mathews, K. G., Ruigrok, M. S., Mansion, G.. 2015. Phylogeny and Biogeography of the Eastern

North American Rose Gentians (*Sabatia*, Gentianaceae). Systematic Botany 811-825.

Mayden, R. L., Allen, J.. 2015. Phylogeography of *Pteronotropis signipinnis, P. euryzonu*s, and

the *P. hypselopterus* Complex (Teleostei: Cypriniformes), with Comments on Diversity and History of the Gulf and Atlantic Coastal Streams. Biomed Research International 2015:675260.

McCarthy, D. M., Mason-Gamer, R. J.. 2016. Chloroplast DNA-Based Phylogeography of *Tilia*

*americana* (Malvaceae). Systematic Botany 41:865-880.

McKay, B. D.. 2009. Evolutionary History Suggests Rapid Differentiation in the Yellow-

Throated Warbler *Dendroica dominica*. Journal of Avian Biology 40:181-190.

McKelvy, A. D., Burbrink, F. T.. 2017. Ecological Divergence in the Yellow-Bellied Kingsnake

(*Lampropeltis calligaster*) At Two North American Biodiversity Hotspots. Molecular Phylogenetics and Evolution 106:61-72.

Merz, C., Catchen, J. M., Hanson-Smith, V., Emerson, K. J., Bradshaw, W. E., Holzapfel, C. M..

2013. Replicate Phylogenies and Post-Glacial Range Expansion of the Pitcher-Plant Mosquito, *Wyeomyia smithii,* in North America. PLoS ONE 8:72262.

Mila, B., Van Tassell, J. L., Calderon, J. A., Ruber, L., Zardoya, R.. 2017. Cryptic Lineage

Divergence in Marine Environments: Genetic Differentiation At Multiple Spatial and Temporal Scales in the Widespread Intertidal goby *Gobiosoma bosc*. Ecology and Evolution 7:5514-5523.

Mobley, K. B., Small, C. M., Jue, N. K., Jones, A. G.. 2010. Population Structure of the Dusky

Pipefish (*Syngnathus floridae*) from the Atlantic and Gulf of Mexico, as Revealed By Mitochondrial DNA and Microsatellite Analyses. Journal of Biogeography 37:1363-1377.

Moncrief, N. D., Lack, J. B., Maldonado, J. E., Bryant, K. L., Edwards, C. W., Van Den

Bussche, R. A.. 2012. General Lack of Phylogeographic Structure in Two Sympatric, Forest Obligate Squirrels (*Sciurus niger and S. carolinensis*). Journal of Mammalogy 93:1247-1264.

Moncrief, N. D., Lack, J. B., Van Den Bussche, R. A.. 2010. Eastern Fox Squirrel (*Sciurus*

*niger*) Lacks Phylogeographic Structure: Recent Range Expansion and Phenotypic Differentiation. Journal of Mammalogy 91:1112-1123.

Morris, A. B., Graham, C. H., Soltis, D. E., Soltis, P. S.. 2010. Reassessment of

Phylogeographical Structure in an Eastern North American Tree Using Monmonier's Algorithm and Ecological Niche Modelling. Journal of Biogeography 37:1657-1667.

Morris, A. B., Ickert-Bond, S. M., Brunson, D. B., Soltis, D. E., Soltis, P. S.. 2008.

Phylogeographical Structure and Temporal Complexity in American Sweetgum (*Liquidambar styraciflua*, Altingiaceae). Molecular Ecology 17:3889-3900.

Nagoshi, R. N., Meagher, R. L., Hay-Roe, M.. 2012. Inferring the Annual Migration Patterns of

Fall Armyworm (Lepidoptera: Noctuidae) in the United States from Mitochondrial Haplotypes. Ecology and Evolution 2:1458-1467.

Nagoshi, R. N., Meagher, R. L., Hay-Roe, M.. 2014. Assessing the Resolution of Haplotype

Distributions to Delineate Fall Armyworm (Lepidoptera: Noctuidae) Migratory Behaviors. Journal of Economic Entomology 107”1462-1470.

Newman, C. E., Austin, C. C.. 2015. Thriving in the Cold: Glacial Expansion and Post-Glacial

Contraction of a Temperate Terrestrial Salamander (*Plethodon serratus*). PLoS ONE 10: 0130131.

Newman, C. E., Rissler, L. J.. 2011. Phylogeographic analyses of the Southern Leopard Frog:

The Impact of Geography and Climate on the Distribution of Genetic Lineages Vs. Subspecies. Molecular Ecology 20:5295-5312.

Ney, G., Schul, J.. 2017. Population Structure within the One-Dimensional Range of a Coastal

Plain Katydid. PLoS ONE 12: 0179361.

Oliveira, L. O., Huck, R. B., Gitzendanner, M. A., Judd, W. S., Soltis, D. E., Soltis, P. S.. 2007.

Molecular Phylogeny, Biogeography, and Systematics of *Dicerandra* (Lamiaceae), a Genus Endemic to the Southeastern United States. American Journal of Botany 94:1017-1027.

Oswald, K. J., Grady, J. M., Quattro, J. M.. 2009. Cytonuclear Patterns of Genetic Diversity and

the Intricate Evolutionary History of the Inland Silverside (*Menidia beryllina*). Journal of Heredity 100:526-532.

Padhi, A.. 2012. Mitochondrial DNA Evidence for Late Pleistocene Population Expansion of the

White Catfish, *Ameiurus catus*. Biochemical Systematics and Ecology 42:94-98.

Padhi, A.. 2013. Contrasting Patterns of Genetic Diversity Between the Northern and Southern

Populations of Yellow Bullhead Catfish, *Ameiurus natalis* in North America. Aquatic Ecology 347:57-363.

Padhi, A.. 2013. Genetic Evidence of Multiple Matrilineal Lineages of the Channel Catfish,

*Ictalurus punctatus* in North America. Conservation Genetics 14:907-912.

Padhi, Abinash. 2014. Geographic Variation Within a Tandemly Repeated Mitochondrial DNA

D-Loop Region of a North American Freshwater Fish, *Pylodictis olivaris*. Gene 538:63-68.

Park, B., Donoghue, M. J.. 2019. Phylogeography of a Widespread Eastern North American

Shrub, *Viburnum lantanoides*. American Journal of Botany 106:389-401

Pauly, G. B., Bennett, S. H., Palis, J. G., Shaffer, H. B.. 2012. Conservation and Genetics of the

Frosted Flatwoods Salamander (*Ambystoma cingulatum*) on the Atlantic Coastal Plain. Conservation Genetics 13:1-7.

Pessino, M., Chabot, E. T., Giordano, R. A, Dewalt, R. E.. 2014. Refugia and Postglacial

Expansion of *Acroneuria frisoni* Stark & Brown (Plecoptera: Perlidae) in North America. Freshwater Science 33:232-249.

Peterson, B. J., Graves, W. R.. 2016. Chloroplast Phylogeography of *Dirca palustris* L. Indicates

Populations Near the Glacial Boundary at the Last Glacial Maximum in Eastern North America. Journal of Biogeography 43:314-327.

Pfeiffer, J. M., Sharpe, A. E., Johnson, N. A., Emery, K. F., Page, L. M.. 2018. Molecular

Phylogeny of the Nearctic and Mesoamerican Freshwater Mussel Genus *Megalonaias*. Hydrobiologia 811:139-151

Piller, K. R., Bart, H. L., Jr., Hurley, D. L.. 2008. Phylogeography of the Greenside Darter

Complex, *Etheostoma blennioides* (Teleostomi: Percidae): a Wide-Ranging Polytypic Taxon. Molecular Phylogenetics and Evolution 46:974-985.

Puckett, E. E., Etter, P. D., Johnson, E. A., Eggert, L. S.. 2015. Phylogeographic analyses of

American Black Bears (*Ursus americanus*) Suggest Four Glacial Refugia and Complex Patterns of Postglacial Admixture. Molecular Biology and Evolution 32:2338-2350.

Pyron, R. A., Burbrink, F. T.. 2009. Lineage Diversification in a Widespread Species: Roles for

Niche Divergence and Conservatism in the Common Kingsnake, *Lampropeltis getula*. Molecular Ecology 18:3443-3457.

Pyron, R. A., Hsieh, F. W., Lemmon, A. R., Lemmon, E. M., Hendry, C. R.. 2016. Integrating

Phylogenomic and Morphological Data to Assess Candidate Species-Delimitation Models in Brown and Red-Bellied Snakes (*Storeria*). Zoological Journal of the Linnean Society 177:937-949.

Ramaiya, M., Johnson, M. G., Shaw, B., Heinrichs, J., Hentschel, J., Von Konrat, M., Davison,

P. G., Shaw, A. J.. 2010. Morphologically Cryptic Biological Species Within the Liverwort *Frullania asagrayana*. American Journal of Botany 97:1707-1718.

Richter, S. C., O'Neill, E. M., Nunziata, S. O., Rumments, A., Gustin, E. S., Young, J. E.,

Crother, B. I.. 2014. Cryptic Diversity and Conservation of Gopher Frogs Across the Southeastern United States. Copeia 2:231-237.

Rodrigues, A., Stefanovic, S.. 2016. Present-Day Genetic Structure of the Holoparasite

Conopholis Americana (Orobanchaceae) in Eastern North America and the Location of its Refugia During the Last Glacial Cycle. International Journal of Plant Sciences 177:132-144.

Rubinoff, D., San Jose, M., Peigler, R. S.. 2017. Multi-Gene Phylogeny of the *Hemileuca maia*

Complex (Saturniidae) Across North America Suggests Complex Phylogeography and Rapid Ecological Diversification. Systematic Entomology 42:621-634

Saeki, I., Dick, C.W., Barnes, B. V., Murakami, N.. 2011. Comparative Phylogeography of Red

Maple (*Acer rubrum* L.) and Silver Maple (*Acer saccharinum* L.): Impacts of Habitat Specialization, Hybridization and Glacial History. Journal of Biogeography 38:992-1005.

Sakata, Y., Itami, J., Isagi, Y., Ohgushi, T.. 2015. Multiple and Mass Introductions from Limited

Origins: Genetic Diversity and Structure of *Solidago altissima* in the Native and Invaded Range. Journal of Plant Research 128:909-921.

Sanchez-Ramirez, S., Tulloss, R. E., Guzman-Davalos, L., Cifuentes-Blanco, J., Valenzuela,

Ricardo, Estrada-Torres, A., Ruan-Soto, F., Diaz-Moreno, R., Hernandez-Rico, N., Torres-Gomez, M., Leon, H., Moncalvo, Jean-Marc. 2015. In and Out of Refugia: Historical Patterns of Diversity and Demography in the North American Caesar's Mushroom Species Complex. Molecular Ecology 24:5938-5956.

Satler, J. D., Carstens, B. C.. 2016. Phylogeographic Concordance Factors Quantify

Phylogeographic Congruence Among Co-Distributed Species in the *Sarracenia alata* Pitcher Plant System. Evolution 170:105-1119.

Satler, J. D., Carstens, B. C.. 2017. Do Ecological Communities Disperse Across Biogeographic

Barriers as a Unit?. Molecular Ecology 26:3533-3545.

Schonhuth, S., Gagne, R. B., Alda, F., Neely, D.A., Mayden, R. L., Blum, M. J.. 2018.

Phylogeography of the Widespread Creek Chub *Semotilus atromaculatus* (Cypriniformes: Leuciscidae). Journal of Fish Biology 93:778-791

Schrey, N. M., Schrey, A. W., Heist, E. J., Reeve, J. D.. 2011. Genetic Heterogeneity in a

Cyclical Forest Pest, the Southern Pine Beetle, *Dendroctonus frontalis*, is Differentiated into East and West Groups in the Southeastern United States. Journal of Insect Science 11:110.

Scott, P. A., Glenn, T. C., Rissler, L. J.. 2018. Resolving Taxonomic Turbulence and Uncovering

Cryptic Diversity in the Musk Turtles (*Sternotherus*) Using Robust Demographic Modeling. Molecular Phylogenetics and Evolution 120:1-15

Seal, J. N., Brown, L., Ontiveros, C., Thiebaud, J., Mueller, U. G.. 2015. Gone to Texas:

Phylogeography of Two *Trachymyrmex* (Hymenoptera: Formicidae) Species Along the Southeastern Coastal Plain of North America. Biological Journal of the Linnean Society 114:689-698.

Shaffer, A. A., Dowler, R. C., Perkins, J. C., Ferguson, A. W., Mcdonough, M. M., Ammerman,

L. K.. 2018. Genetic Variation in the Eastern Spotted Skunk (*Spilogale putorius*) with Emphasis on the Plains Spotted Skunk (*S. p. interrupta*). Journal of Mammalogy 99:1237-1248

Shaw, J., Craddock, J. H., Binkley, M. A.. 2012. Phylogeny and Phylogeography of North

American Castanea Mill. (Fagaceae) Using cpDNA Suggests Gene Sharing in the Southern Appalachians (Castanea Mill., Fagaceae). Castanea 77:186-211.

Simonsen, T. J., Brown, R. L., Sperling, F. A. H.. 2008. Tracing an Invasion: Phylogeography of

*Cactoblastis cactorum* (Lepidoptera : Pyralidae) in the United States Based on Mitochondrial DNA. Annals of the Entomological Society of America 101:899-905.

Soto-Centeno, J. A, Barrow, L. N., Allen, J. M., Reed, D. L.. 2013. Reevaluation of a Classic

Phylogeographic Barrier: New Techniques Reveal the Influence of Microgeographic Climate Variation on Population Divergence. Ecology and Evolution 3:1603-1613.

Spriggs, E. L., Eaton, D. A. R., Sweeney, P. W., Schlutius, C., Edwards, E. J., Donoghue, M. J..

2019. Restriction-Site-Associated DNA Sequencing Reveals a Cryptic *Viburnum* Species on the North American Coastal Plain. Systematic Biology 68:187-203

Spriggs, E. L., Schlutius, C., Eaton, D. A., Park, B., Sweeney, P. W., Edwards, E. J., Donoghue,

M. J.. 2019. Differences in Flowering Time Maintain Species Boundaries in a Continental Radiation of *Viburnum*. American Journal of Botany 106:833-849

Stephens, J.D., Rogers, W. L., Heyduk, K., Cruse-Sanders, J. M., Determann, R. O., Glenn, T.

C., Malmberg, R. L.. 2015. Resolving Phylogenetic Relationships of the Recently Radiated Carnivorous Plant Genus *Sarracenia* Using Target Enrichment. Molecular Phylogenetics and Evolution 85:76-87.

Stephens, J. D., Santos, S. R., Folkerts, D. R.. 2011. Genetic Differentiation, Structure, and a

Transition Zone Among Populations of the Pitcher Plant Moth *Exyra semicrocea*: Implications for Conservation. PLoS ONE 6:22658.

Strickland, J. L., Parkinson, C. L., McCoy, J. K., Ammerman, L. K.. 2014. Phylogeography of

*Agkistrodon piscivorus* with Emphasis on the Western Limit of its Range. Copeia 4:639-649.

Szalanski, A. L., Austin, J. W., Mckern, J. A., Scheffrahn, R.H., Owens, C. B., Messenger, M.

T.. 2008. Molecular Phylogeography of *Reticulitermes* (Isoptera : Rhinotermitidae) Termites from Florida. Sociobiology 52:619-632.

Tanaka, A., Ohtani, M., Suyama, Y., Inomata, N., Tsumura, Y., Middleton, B. A., Tachida, H.,

Kusumi, J.. 2012. Population Genetic Structure of a Widespread Coniferous Tree, *Taxodium distichum* [L.] Rich. (Cupressaceae), in the Mississippi River Alluvial Valley and Florida. Tree Genetics & Genomes 8:1135-1147.

Thesing, B. D., Noyes, R. D., Starkey, D. E., Shepard, D. B.. 2016. Pleistocene Climatic

Fluctuations Explain the Disjunct Distribution and Complex Phylogeographic Structure of the Southern Red-Backed Salamander, *Plethodon serratus*. Evolutionary Ecology 30:89-104.

Tollis, M., Boissinot, S.. 2014. Genetic Variation in the Green anole Lizard (*Anolis carolinensis*)

Reveals Island Refugia and a Fragmented Florida During the Quaternary. Genetica 142:59-72.

Tollis, M., Ausubel, G., Ghimire, D., Boissinot, S.. 2012. Multi-Locus Phylogeographic and

Population Genetic analysis of *Anolis carolinensis*: Historical Demography of a Genomic Model Species. PLoS ONE 7: 38474.

Trapnell, D. W., Schmidt, J. P., Quintana-Ascencio, P. F., Hamrick, J. L.. 2007. Genetic Insights

into the Biogeography of the Southeastern North American Endemic, *Ceratiola ericoides* (Empetraceae). Journal of Heredity 98”587-593.

Triplett, J. K., Oltrogge, K. A., Clark, L. G.. 2010. Phylogenetic Relationships and Natural

Hybridization Among the North American Woody Bamboos (Poaceae: Bambusoideae: *Arundinaria*). American Journal of Botany 97:471-492.

Tursi, R. M., Hughes, P. T., Hoffman, E. A.. 2013. Taxonomy Versus Phylogeny: Evolutionary

History of Marsh Rabbits Without Hopping to Conclusions. Diversity and Distributions 19:120-133.

Vargas-Rodriguez, Y. L., Platt, W. J., Urbatsch, L. E., Foltz, D. W.. 2015. Large Scale Patterns

of Genetic Variation and Differentiation in Sugar Maple from Tropical Central America to Temperate North America. BMC Evolutionary Biology 15:257.

Vickruck, J. L., Richards, M. H.. 2017. Nesting Habits Influence Population Genetic Structure of

a Bee Living in anthropogenic Disturbance. Molecular Ecology 26:2674-2686.

Vonhof, M. J., Russell, A. L.. 2015. Genetic Approaches to the Conservation of Migratory Bats:

A Study of the Eastern Red Bat (*Lasiurus borealis*). PeerJ 3:983.

Walker, M. J., Stockman, A. K., Marek, P. E., Bond, J. E.. 2009. Pleistocene Glacial Refugia

Across the Appalachian Mountains and Coastal Plain in the Millipede Genus *Narceus*: Evidence from Population Genetic, Phylogeographic, and Paleoclimatic Data. BMC Evolutionary Biology 9:25.

Wallace, L. E., Doffitt, C. H.. 2013. Genetic Structure of the Mesic Forest-Adapted Herbs

*Trillium cuneatum* and *Trillium stamineum* (Melanthiaceae) in the South-Central United States. Castanea 78:154-162.

Weinell, J. L., Austin, C. C.. 2017. Refugia and Speciation in North American Scarlet Snakes

(*Cemophora*). Journal of Herpetology 51:161-171.

Whitehead, A.. 2010. the Evolutionary Radiation of Diverse Osmotolerant Physiologies in

Killifish (*Fundulus* sp.). Evolution 64:2070-2085.

Williams, D. A., Brown, S. D., Crawford, D. L.. 2008. Contemporary and Historical Influences

on the Genetic Structure of the Estuarine-Dependent Gulf Killifish *Fundulus grandis*. Marine Ecology Progress Series 373:111-121.

Williford, D., Deyoung, R. W., Honeycutt, R. L., Brennan, L. A., Hernandez, F.. 2016.

Phylogeography of the Bobwhite (*Colinus*) Quails. Wildlife Monographs 193:1-49.

Zanatta, D. T., Murphy, R. W.. 2008. the Phylogeographical and Management Implications of

Genetic Population Structure in the Imperiled Snuffbox Mussel, *Epioblasma triquetra* (Bivalvia : Unionidae). Biological Journal of the Linnean Society 93:371-384.

Zellmer, A. J., Hanes, M. M., Hird, S. M., Carstens, B. C.. 2012. Deep Phylogeographic

Structure and Environmental Differentiation in the Carnivorous Plant *Sarracenia alata*. Systematic Biology 61:763-777.

Zhou, W., Ji, X., Obata, S. Pais, A., Dong, Y., Peet, R., Xiang, Qiu-Yun (Jenny). 2018.

Resolving Relationships and Phylogeographic History of the *Nyssa sylvatica* Complex Using Data from RAD-Seq and Species Distribution Modeling. Molecular Phylogenetics and Evolution 126:1-16.
